# Beyond Compliance: Redefining Food Quality and Safety from Hippocratic Dietetics to Food Exposomics, Chemical Mixtures and Future Food Systems

**DOI:** 10.3390/foods15213969

**Published:** 2026-11-05

**Authors:** Radosław Kowalski, Grażyna Kowalska

**Affiliations:** 1 Department of Analysis and Food Quality Assessment, University of Life Sciences in Lublin, 20-704 Lublin, Poland; 2 Department of Tourism and Recreation, University of Life Sciences in Lublin, 20-950 Lublin, Poland

**Keywords:** food quality, food safety, chemical mixtures, cumulative risk assessment, chemical exposure, food exposomics, foodomics, emerging contaminants, public health, future food systems

## Abstract

Food quality has traditionally been assessed through compliance with legal requirements, microbiological and chemical safety, nutritional value, and control of individual contaminants. Contemporary food systems, however, require a broader perspective that also considers matrix complexity, processing, chemical mixtures, long-term exposure, emerging technologies, and variation in consumer responses. This narrative-conceptual review, supported by a structured literature search, reframes food quality from a *beyond compliance* perspective and develops three related propositions. *Food quality under uncertainty* addresses incomplete knowledge concerning the chemical space of food, mixture effects, actual exposure, and emerging technologies. *Sub-threshold multi-contaminant burden* describes recurrent co-exposure profiles in which individual contaminants remain below their regulatory limits, without implying inherent harm, but highlighting the need for improved monitoring, modeling, and cumulative exposure assessment. The *New Food Quality Compass* organizes quality as a multidimensional profile integrating safety, nutritional value, bioactivity, processing, contaminants, authenticity, sustainability, personalization, and trust. Future assessment should combine targeted, suspect, and non-target analysis with foodomics, exposomics, consumption data, and risk–benefit interpretation. The *beyond compliance* perspective extends conventional safety control toward a more integrated framework linking food composition, chemical exposure, uncertainty, public health, and future food systems.

## 1. Introduction

The concept of food quality needs to be reconsidered because conventional assessment of product compliance with legal requirements no longer captures the full complexity of the relationships among food, human health, technology, the environment, and public trust. Historically, food was viewed as an element of health maintenance and dietary practice, but the popular phrase “let food be thy medicine” should not be attributed to Hippocrates as an authentic quotation. It is more appropriate to refer to the Hippocratic tradition as one of the historical foundations of thinking about diet, lifestyle, and prevention rather than as a direct equation of food with medicine [1].

Modern food safety systems developed in response to tangible hazards, including poisoning, microbiological contamination, natural toxins, food adulteration, and the consequences of increasingly globalized supply chains. World Health Organization (WHO) estimates indicate that foodborne diseases continue to impose a substantial public-health burden worldwide and involve microbiological, parasitic, and chemical hazards [2]. Conventional official control remains fundamental to food safety, but systems based on regulatory limits, monitoring, and targeted analyses need to be complemented by tools capable of interpreting complex patterns of exposure and risk [3].

For many decades, food quality assessment has been organized around the logic of compliance: a product was considered acceptable if it met specified criteria for composition, purity, microbiological safety, and maximum contaminant levels. This approach remains necessary because it enables regulatory enforcement, consumer protection, and comparability of results among laboratories. Its ability to describe quality becomes more limited, however, when a product or the diet as a whole contains numerous compounds occurring simultaneously at low levels. The European Food Safety Authority (EFSA) has developed a harmonized approach to the assessment of combined exposure to multiple chemicals, while the literature on pesticide residues points to the need for further development of cumulative and probabilistic risk models [3,4,5]. The broader toxicological literature likewise emphasizes that regulations based primarily on single-substance assessment may be insufficient when actual patterns of co-exposure are considered [6,7]. Compliance with requirements established for individual substances therefore does not fully characterize the chemical and exposure profile of a product, particularly when residues, metabolites, or substances originating from processing and packaging occur together [8,9].

The quality profile of food is shaped not only by the raw material and its composition, but also by the production environment, processing, packaging, storage, transport, culinary preparation, and patterns of consumption. Substances migrating from food-contact materials, including intentionally used compounds, degradation products, and non-intentionally added substances (NIAS), are becoming an increasingly important component of exposure assessment [8,9]. Studies of micro- and nanoplastics originating from food-contact materials and of per- and polyfluoroalkyl substances (PFAS) exposure through the global trade in fish show that the chemical quality of food cannot be considered solely at the local level but must also be viewed in the context of global supply chains [10,11].

Food processing brings both benefits and potential risks. Thermal treatment, fermentation, preservation, extrusion, and packaging can improve microbiological safety, shelf life, accessibility, and the digestibility or bioavailability of selected constituents [12,13,14]. Intensive processing may also alter the food matrix, reduce the stability of sensitive components, promote the formation of processing-derived compounds, increase palatability, and facilitate excessive energy intake [13,14,15,16,17]. The review examines quality trade-offs across different food categories without relying on a simple opposition between “natural” and “technological” foods.

Ultra-processed foods provide an example of a category in which assessment based solely on declared nutrient composition may be insufficient. The NOVA classification drew attention to the possibility that dietary assessment may depend not only on the content of sugar, fat, salt, or energy, but also on the nature, purpose, and extent of technological processing [17]. The umbrella review by Lane et al. linked greater exposure to ultra-processed foods with a range of adverse health outcomes, whereas the controlled study by Hall et al. showed that an ultra-processed diet may increase spontaneous energy intake and body weight even when major nutritional characteristics of the diets are matched [15,16]. These findings indicate that declared composition alone does not always capture the influence of a food product on energy intake and eating behavior.

Food quality also requires a systems-level interpretation. The EAT-Lancet reports place food within the broader context of healthy, sustainable, and equitable food systems [18,19]. Crippa et al. showed that food systems account for a substantial share of global anthropogenic greenhouse gas emissions, bringing environmental impact, supply-chain resilience, and the long-term costs of production into the discussion of food quality [20]. Climate change may also alter the occurrence patterns of hazards, including mycotoxins, which supports a more anticipatory rather than exclusively reactive approach to food quality monitoring [21].

Within this broader perspective, the concepts of foodomics, the dietary exposome, and food exposomics are becoming increasingly relevant. Targeted analysis remains the basis of compliance control, but it cannot cover the entire chemical space of food, particularly unexpected compounds, metabolites, degradation products, and substances present at trace levels [22,23,24,25]. The dietary exposome approach links data on the occurrence of chemicals in food with consumption patterns and chronic human exposure, while exposure biomarker databases developed within Exposome-Explorer illustrate the value of connecting food analysis with biomonitoring and metabolomics [26,27]. Advances in nutritional metabolomics further suggest that future quality assessment may consider not only product composition but also the biological response of the organism to food intake and chemical exposure [28,29].

Personalization is becoming increasingly relevant to the interpretation of food quality. The same product may have different nutritional value and different safety implications for a healthy person, an individual with metabolic disorders, a different microbiota profile, another lifestyle, or a different background of environmental exposure [28,29,30,31]. Future approaches to food quality should connect product assessment with the diet as a whole, the individual characteristics of the consumer, and data on long-term exposure [26,29,31]. From this perspective, personalized nutrition may be extended to include personalized food safety and exposure interpretation.

New food sources and production systems further broaden the range of questions surrounding quality. Plant-based alternative proteins, edible insects, algae, precision fermentation, cultured meat, controlled-environment agriculture, technologically designed foods, and space foods are being developed, among other reasons, in response to environmental pressures, food-security challenges, and resource constraints [32,33,34,35,36]. Each of these categories nevertheless requires its own quality assessment, ranging from nutritional value and allergenicity to matrix stability, process-derived metabolites, consumer acceptance, cost, regulation, and ethical considerations. Future foods should not be regarded as automatic solutions to existing problems, but as an area requiring timely monitoring and food-safety foresight [34,37].

The aim of this review is to present a conceptual framework for redefining food quality from a *beyond compliance* perspective, extending assessment beyond compliance with legal requirements alone. The review integrates chemical safety, nutritional value, bioactivity, processing, contaminant mixtures, hidden exposures, foodomics, personalization, and emerging food-production systems. On this basis, the framework of *food quality under uncertainty* and the other conceptual elements developed in the following sections are introduced [3,6,26,37].

## 2. Historical and Conceptual Background: From Hippocratic Dietetics to Industrial Food Systems

Thinking about food quality has changed in response to health, social, and technological needs rather than through a simple progression from “natural” to “industrial” food. In the Hippocratic tradition, diet formed part of a broader approach to health that included lifestyle, balance, and adaptation of diet to the individual; however, this tradition should not be reduced to the popular notion of food as medicine [1]. As awareness of hazards grew, food quality also came to encompass protection against poisoning, natural toxins, spoilage, and adulteration and, more recently, issues associated with global supply chains, food fraud, and intentional or unintentional changes in composition [2,38,39].

Industrialization added a clear technological and institutional dimension to quality assessment. Processing, preservation, refrigeration, packaging, standardization, and laboratory control improved food availability and shelf life and reduced many microbiological hazards [12,40]. At the same time, consumers increasingly came to rely on labels, certification, institutions, and producers rather than direct knowledge of food origin [38,39,40]. Contemporary quality assessment therefore extends beyond availability, shelf life, and product safety to include health, environmental, and consumption-related dimensions [40,41].

The present stage can be described as systemic and exposure-oriented. Food quality is no longer only a property of an individual product, but the outcome of the entire chain, including primary production, the environment, processing, packaging, transport, trade, preparation, and actual consumption patterns [18,19,20,41]. From this perspective, safety, nutritional value, bioactivity, authenticity, environmental footprint, economic accessibility, and public trust jointly shape a quality profile that cannot be reduced to a single parameter [18,19,20,41]. In this context, *food quality under uncertainty* refers to quality assessment conducted under incomplete knowledge of the product’s chemical space, mixture effects, and the consequences of emerging technologies [3,6,9,10,34]. This view is closely related to the concept of *risk governance*, which accounts for the complexity and uncertainty of risk, the involvement of multiple actors in decision-making, and its social dimension [42].

Compliance assessment addresses whether legal requirements are met, whereas a broader interpretation of quality also considers the profile of benefits, risks, and uncertainties under actual patterns of consumption [3,4,5,6,42]. 

**Figure 1:**
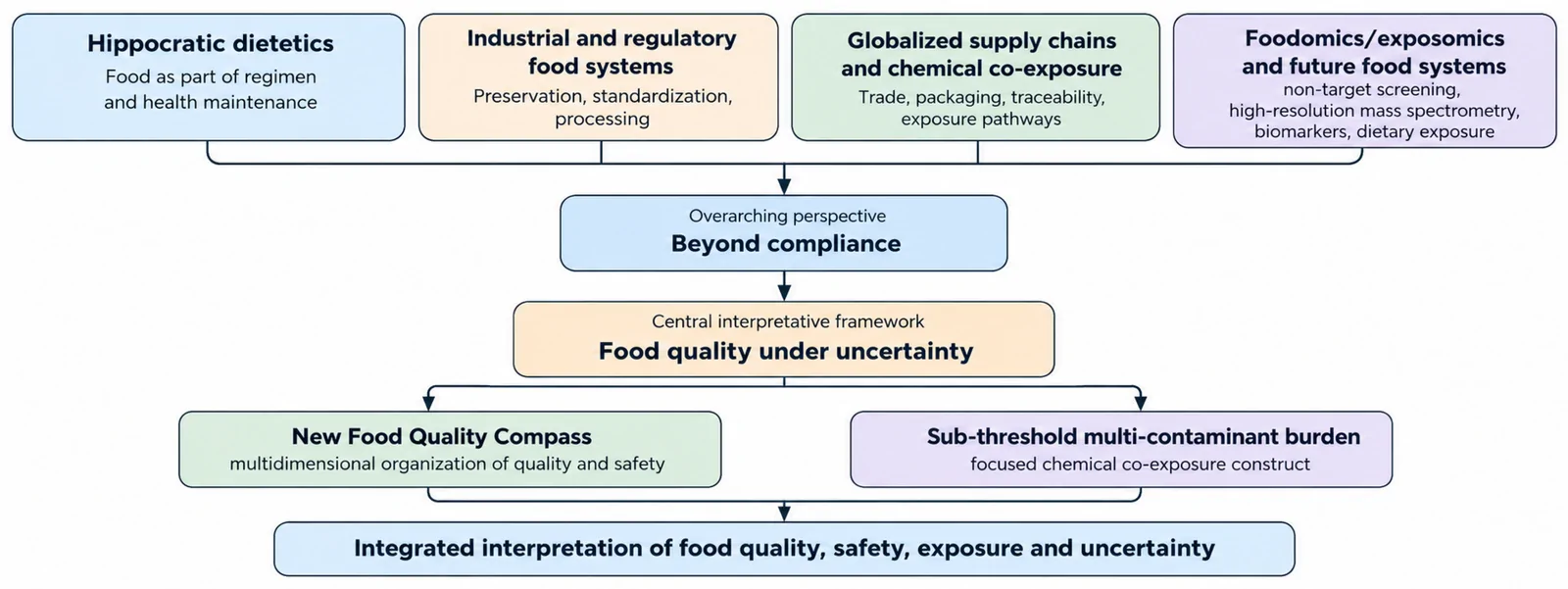
Hierarchical structure of the conceptual framework. The beyond-compliance perspective is interpreted through food quality under uncertainty, with the New Food Quality Compass organizing the broader dimensions of quality and safety and sub-threshold multi-contaminant burden addressing chemical co-exposure more specifically.

Figure 1 brings together two aspects of the review: the changing context in which food has been assessed and the relationships among the main concepts developed here. The shift from dietetic views of food and industrial and regulatory control systems [1,3,40] toward approaches that also address chemical mixtures, foodomics/exposomics, and future food systems [22,26,34] provides the background for the beyond-compliance perspective. Food quality under uncertainty forms its main interpretive frame. The New Food Quality Compass and sub-threshold multi-contaminant burden then address different parts of that frame: the Compass organizes the broader dimensions of quality and safety, whereas the burden concept focuses more specifically on recurrent chemical co-exposure.

## 3. Methods and Literature Search Strategy

The review was prepared as a narrative-conceptual synthesis supported by a structured literature search. Its design and underlying assumptions were considered in relation to established typologies of narrative and integrative reviews [43,44]. Preferred Reporting Items for Systematic Reviews and Meta-Analyses (PRISMA), its search-reporting extension (PRISMA-S), and the PRISMA extension for scoping reviews (PRISMA-ScR) recommendations were used only as supporting references for transparent reporting of the search process, without claiming compliance with a full systematic-review protocol [45,46,47]. The quality of the narrative synthesis and use of evidence was additionally considered in relation to Scale for the Assessment of Narrative Review Articles (SANRA) and EFSA guidance [48,49].

### 3.1. Review Design and Rationale

The methodological basis of this work is a narrative-conceptual review focused on synthesizing trends, research gaps, and directions for further development rather than simply summarizing findings from a single narrow field. This approach was chosen because of the broad scope of the topic, which includes food chemistry, mixture toxicology, instrumental analysis, sample preparation, ultra-processed foods, future foods, food policy, education, and risk communication [43,44,48].

Literature selection was organized at three levels. The first included studies providing conceptual frameworks for areas such as food quality, food safety, food systems, processing, ultra-processed foods, foodomics, exposomics, and future foods. The second covered mechanistic and methodological publications on chemical mixtures, exposure assessment, suspect and non-target screening, sample preparation, and emerging analytical technologies. The third comprised institutional and foresight-oriented documents, particularly from EFSA, Food and Agriculture Organization of the United Nations (FAO), and WHO, because of their importance in food safety risk assessment, method harmonization, and the development of public policy [3,34,37,49].

### 3.2. Information Sources and Time Frame

The literature search was conducted primarily in Web of Science Core Collection, Scopus, and PubMed/MEDLINE, with ScienceDirect and Google Scholar used as supplementary sources. Web of Science and Scopus were treated as the main interdisciplinary databases, PubMed/MEDLINE as a source of biomedical, toxicological, and nutritional literature, and ScienceDirect as a supplementary source for publications in food technology, food chemistry, and analytical science. Google Scholar was used to identify backward and forward citations, institutional reports, and publications that were less readily indexed, but it was not used as the sole search source [50,51,52].

The main search period covered 2000–2026. Older publications were included selectively when they were fundamental to the historical development of concepts, food technology, classical analytical methods, or review methodology [1,5,43,53]. The bibliography was updated with particular attention to rapidly developing areas, including PFAS, micro- and nanoplastics, food-contact chemicals, future foods, artificial intelligence (AI), and suspect and non-target screening. The final search update was conducted in October 2026.

### 3.3. Search Strategy and Keyword Blocks

The search strategy was based on thematic blocks combined using the Boolean operators AND/OR. The first block covered general concepts related to food quality and safety: “food quality”, “food safety”, “food systems”, “food chain”, “field to fork”, “food policy”, “risk governance”, and “food safety foresight”. The second block addressed contaminants and exposure: “food contaminants”, “chemical mixtures”, “cumulative risk assessment”, “combined exposure”, “dietary exposure”, “food exposome”, “dietary exposome”, “PFAS”, “microplastics”, “nanoplastics”, “mycotoxins”, “process contaminants”, “food contact materials”, “food contact chemicals”, and “NIAS”.

The third block covered processing and food categories: “minimally processed foods”, “traditional foods”, “organic foods”, “conventional foods”, “ultra-processed foods”, “NOVA classification”, “food matrix”, “food processing”, “extrusion”, “fermentation”, “thermal processing”, and “process-induced contaminants”. The fourth block focused on analytical tools: “targeted analysis”, “suspect screening”, “non-target screening”, “liquid chromatography-high-resolution mass spectrometry (LC-HRMS)”, “gas chromatography-high-resolution mass spectrometry (GC-HRMS)”, “foodomics”, “metabolomics”, “chemometrics”, “sample preparation”, “quick, easy, cheap, effective, rugged, and safe (QuEChERS)”, “micro-QuEChERS”, “green analytical chemistry”, “hyperspectral imaging”, “Raman”, “surface-enhanced Raman spectroscopy (SERS)”, and “biosensors” [53,54,55,56,57].

The fifth block addressed future food sources and personalization: “future foods”, “novel foods”, “alternative proteins”, “edible insects”, “algae”, “precision fermentation”, “cultured meat”, “space food”, “personalized nutrition”, “artificial intelligence dietary recommendations”, “personalized food safety”, and “risk communication”. Searches were carried out by combining blocks, for example: (“chemical mixtures” OR “combined exposure”) AND (“food” OR “dietary exposure”), (“non-target screening” OR “suspect screening”) AND (“food safety” OR “food contaminants”), and (“future foods” OR “novel foods”) AND (“safety” OR “risk assessment” OR “allergenicity”).

### 3.4. Eligibility Criteria and Publication Prioritization

The review included primarily peer-reviewed review articles and experimental articles, methodological papers, expert statements, EFSA/FAO/WHO documents, and peer-reviewed publications in food science, toxicology, analytical chemistry, nutrition, and food policy. Preference was given to publications with complete bibliographic information, a DOI, clearly described methods, and a direct relationship to at least one of the main dimensions addressed in the manuscript: safety, nutritional value, processing, chemical mixtures, foodomics, sample preparation, future foods, or food-safety foresight.

Studies without a direct connection to the topic were excluded, as were purely popular-science publications, marketing materials, sources for which authorship or publication date could not be identified, and articles lacking basic information on research methods or analytical scope. Older studies were retained only when they had historical or methodological importance or served as reference points for the development of a particular area, such as QuEChERS, review methodology, food classification, or the historical interpretation of the relationship between food and health [1,43,53].

For rapidly developing topics, including chemicals migrating from food-contact materials, PFAS, micro- and nanoplastics, future foods, artificial intelligence, and suspect and non-target screening, recent publications were given priority, particularly those published between 2020 and 2026 and available as peer-reviewed papers or reports from recognized institutions. We did not apply the same five-year cutoff to every topic because the fields covered by this review differ substantially in maturity and rate of development. Older publications were retained when they represented foundational work, original concepts, reference methods, or important methodological landmarks. This was the case, for example, for methodological work on toxicological reviews [58,59]. Where appropriate, these older sources were considered together with more recent reviews, updates, or institutional documents so that the evidence base reflected both the development of the field and its current state. The main elements of the search strategy and inclusion criteria are summarized in Table 1.

### 3.5. Screening, Extraction and Organization of Evidence

Publication selection was conducted in stages. Titles and abstracts were first screened for relevance to the scope of the review. Full texts of potentially relevant publications were then examined. Backward and forward citation searching was also used, particularly for key studies, institutional documents, and publications defining important concepts or methods [50,51].

For each relevant publication, information was extracted on the type of food or production system, the hazard or quality indicator considered, groups of contaminants or bioactive compounds, the analytical method applied, sample-preparation procedures, the approach to exposure assessment, relevance to food policy, and potential research gaps. This approach allowed publications of different types to be integrated within a common interpretive framework.

The evidence was organized thematically rather than purely chronologically. The main groups of literature synthesized corresponded to the structure of the manuscript: multidimensional food quality, paradoxes associated with food categories, contaminant mixtures, hidden exposures, analytical transformation, sample preparation, future foods, space food, risk personalization, education, and food policy.

### 3.6. Synthesis Approach and Conceptual Outputs

The synthesis was qualitative and conceptual. No meta-analysis was performed because the publications considered differed in data type, food matrices, contaminant groups, analytical methods, and levels of interpretation, ranging from product composition and dietary exposure to food policy [43,44,58,59].

Three related concepts emerged from the synthesis, but they are not intended to operate at the same level. *Food quality under uncertainty* provides the broad interpretive frame for situations in which knowledge of the chemical space of food, mixture effects, long-term exposure, or the consequences of emerging production technologies remains incomplete. *The New Food Quality Compass* organizes this broader assessment across ten interdependent dimensions: safety, nutrition, bioactivity, matrix, processing, contaminants, authenticity, sustainability, personalization, and trust. *Sub-threshold multi-contaminant burden* is more specific. It addresses recurrent co-exposure to multiple contaminants that individually occur below their respective threshold values. Although this concept relates most directly to contaminants and safety, its interpretation also depends on consumption patterns and consumer susceptibility. The three concepts are therefore complementary rather than parallel or competing models within the broader *beyond-compliance* perspective.

The synthesis was conducted with interpretive caution. The review does not assume that all natural foods are safe, that all technologically produced foods are risky, or that every mixture of compounds present below regulatory limits constitutes a hazard. Its purpose is to identify where the classical compliance model remains sufficient and where additional analytical, toxicological, educational, and regulatory tools may be needed [3,6,7,42].

### 3.7. Methodological Limitations

The principal methodological limitation is the breadth of the topic. The review spans several disciplines and therefore cannot replace specialized systematic reviews devoted to individual contaminant groups, a single analytical technology, one food category, or one health outcome. Its purpose is instead to integrate perspectives and identify research directions connecting safety, nutritional value, technology, analytical science, exposure, and food policy [43,44]. 

A second limitation is the uneven maturity of the different fields covered. Some areas, such as pesticide residues, QuEChERS, and ultra-processed foods, are supported by a large body of research and review literature. Others, including personalized food safety, *sub-threshold multi-contaminant burden*, food exposomics, and the safety of certain future foods, are still developing. Conclusions were formulated by distinguishing established knowledge from hypotheses requiring further verification and from areas that remain primarily foresight-oriented [22,26,31,34,37].

## 4. Multidimensional Food Quality: Foundations of the New Food Quality Compass

Food quality is treated in this manuscript as a multidimensional concept rather than as a single property of a product. Classical quality assessment has often focused on compliance with requirements, microbiological and chemical safety, nutritional value, shelf life, and sensory acceptability, whereas contemporary food systems call for a considerably broader perspective [60,61]. The same product may be legally safe, sensorially attractive, and technologically stable while showing a less favorable profile in other dimensions, such as the degree of processing, environmental impact, authenticity, long-term exposure, or consumer trust.

In this review, food quality is considered to comprise interdependent dimensions: chemical and microbiological safety, nutritional value, bioactivity, the food matrix, the degree and nature of processing, contaminants, authenticity and traceability, environmental sustainability, personalization, and public trust. Such an approach helps avoid reductionism in which quality is equated solely with the absence of regulatory exceedances, the content of a few nutrients, or the marketing category of “naturalness” [60,61,62].

### 4.1. Safety as a Necessary but Insufficient Condition

Safety remains a fundamental condition of food quality because a product that poses a health risk cannot be regarded as high-quality food, even if it has a favorable nutritional composition or high sensory value [2,60]. Contemporary food safety, however, encompasses not only classical microbiological hazards but also natural toxins, pesticide residues, heavy metals, mycotoxins, process contaminants, substances migrating from food-contact materials, and contaminants associated with the globalization of supply chains [2,3,9,10,11].

Yet safety understood as compliance with an established limit for an individual compound does not provide a complete interpretation of quality. With long-term food consumption, the co-occurrence of many substances at low levels, and the possibility of cumulative dietary exposure, quality assessment should also consider chemical mixtures and actual patterns of consumption [3,4,5,6,7]. Safety is necessary but not sufficient: it answers whether a product falls within the boundaries of acceptable risk, but not necessarily whether its overall profile is optimal for health and the food system.

### 4.2. Nutritional Value, Food Matrix and Processing Context

Nutritional value is one of the most established dimensions of food quality, but it too requires a broader interpretation than a simple sum of protein, fat, carbohydrates, vitamins, and minerals. Studies on processed foods show that such products may simultaneously provide nutrients whose intake should be increased and components whose excessive intake should be limited, including sodium, added sugars, and saturated fatty acids [62]. The category “processed” is not, in itself, an adequate judgment of quality, because composition, technological function, matrix, portion size, and the place of a product within the overall diet all matter [12,62,63].

The food matrix is central to this interpretation. It determines the physical and chemical structure in which food components occur, how they are released during chewing and digestion, how rapidly they become bioavailable, and how they affect the metabolic response [63]. Food should not be interpreted merely as a carrier of isolated constituents; their organization, interactions, degree of comminution, cellular structure, starch gelatinization, protein denaturation, lipid oxidation, and polyphenol binding are also relevant [13,14,63].

From the perspective of food quality, the food matrix concept is closely linked to *food synergy*. Many nutritional effects arise not from the action of a single constituent but from the combined influence of nutrients, bioactive compounds, product structure, and dietary patterns [64]. This view is consistent with more recent concepts of foodomics and food exposomics, which show that the chemical complexity of food extends far beyond the nutrients routinely measured [22,26,65].

### 4.3. Bioactivity and Health-Promoting Potential

Health-promoting potential is often emphasized in research on functional foods and in marketing communication, but its interpretation requires particular caution. The presence of bioactive compounds such as polyphenols, carotenoids, glucosinolates, phytosterols, bioactive peptides, or fermented components does not automatically translate into a documented health effect under real-life consumption conditions [65,66]. Several intermediate levels separate chemical composition from biological effect, including stability during storage and processing, bioaccessibility, bioavailability, metabolism, interactions with the microbiota, and individual biological response [63,66].

Functional foods illustrate this difficulty well. They represent an important area of innovation in the food industry, driven by population aging, rising healthcare costs, and growing interest in nutritional prevention [67]. Their development, however, requires integration of food chemistry, biology, nutrition, pharmacology, statistics, and clinical research, because measurements of bioactive-compound content alone are insufficient to demonstrate genuine health relevance [66]. In this review, bioactivity is treated as a potential dimension of quality only when interpreted in relation to the food matrix, portion size, bioavailability, and the level of supporting evidence [63,65,66].

### 4.4. Authenticity, Traceability and Integrity of the Food Chain

Authenticity is another dimension of quality that becomes increasingly important as supply chains grow more complex. A product may be microbiologically and chemically safe, but if its origin, species or cultivar composition, geographical provenance, or method of production differs from what is declared, its quality and market credibility are compromised [38,68,69]. Food fraud, understood as the intentional adulteration of food for economic gain, may have quality, economic, and ethical consequences and, in some cases, direct health implications [38,39,69].

Authenticity should be regarded not merely as a labeling issue but as a component of safety, trust, and fairness within the food system. Modern authentication techniques include chromatographic, spectroscopic, isotopic, metabolomic, proteomic, and genetic methods, reflecting the need to detect increasingly complex forms of adulteration [68]. Traceability systems are equally important because they allow the history of a product to be reconstructed throughout the supply chain, while molecular tools such as DNA barcoding support the identification of raw materials and species in food [70,71].

### 4.5. Sustainability and Resilience as Dimensions of Quality

Sustainability and resilience extend quality assessment beyond the characteristics of the product itself to the conditions under which food is produced and to the wider food system. The EAT-Lancet reports link human health with sustainable food systems, equity, and planetary boundaries [18,19], while emissions data show that food systems make a substantial contribution to global greenhouse gas emissions [20]. A lower environmental footprint does not, however, make a product automatically safer or nutritionally superior. Assessment should instead make explicit the trade-offs among safety, nutritional value, accessibility, environmental impact, and the resilience of production and supply chains [18,19,20,41,42].

### 4.6. Social Trust, Perception and Communication

Food quality assessment involves not only measurable product characteristics but also how a product is perceived and communicated. Consumer decisions are influenced by labeling, origin, production methods, perceived naturalness, and trust in institutions [61,72]. This is particularly relevant to food additives and emerging technologies, where formal authorization does not necessarily lead to acceptance if consumers remain uncertain about benefits, naturalness, control, ethics, or transparency [72,73,74]. Risk–benefit communication should therefore distinguish clearly between well-established evidence and uncertainty, without minimizing or exaggerating risk [75]. Within the *food quality under uncertainty* framework, trust has a practical role in the functioning of control systems, innovation, and food policy [42,61,75].

### 4.7. New Food Quality Compass

A recent review of Nutri-Score and related front-of-pack systems likewise indicates that nutrient profiling alone does not exhaust the assessment of food quality. Demarquoy describes seven complementary dimensions that include, among others, the food matrix, processing, dietary context, bioactivity, safety and contaminants, sustainability, and individual variability [76]. The present review instead proposes the *New Food Quality Compass*, which organizes food quality as a profile of ten interdependent dimensions rather than as a single numerical indicator. The model includes safety, nutrition, bioactivity, matrix, processing, contaminants, authenticity, sustainability, personalization, and trust.

The Compass does not replace conventional quality control, safety assessment, or dietary recommendations. Its purpose is to organize research and decision-making questions: whether a product is safe, nutritious, stable, authentic, properly described, environmentally responsible, appropriate for a particular consumer, and socially credible. The same product can be examined simultaneously in terms of composition, matrix, chemical exposure, technology, dietary context, trust, and uncertainty.

**Figure 2:**
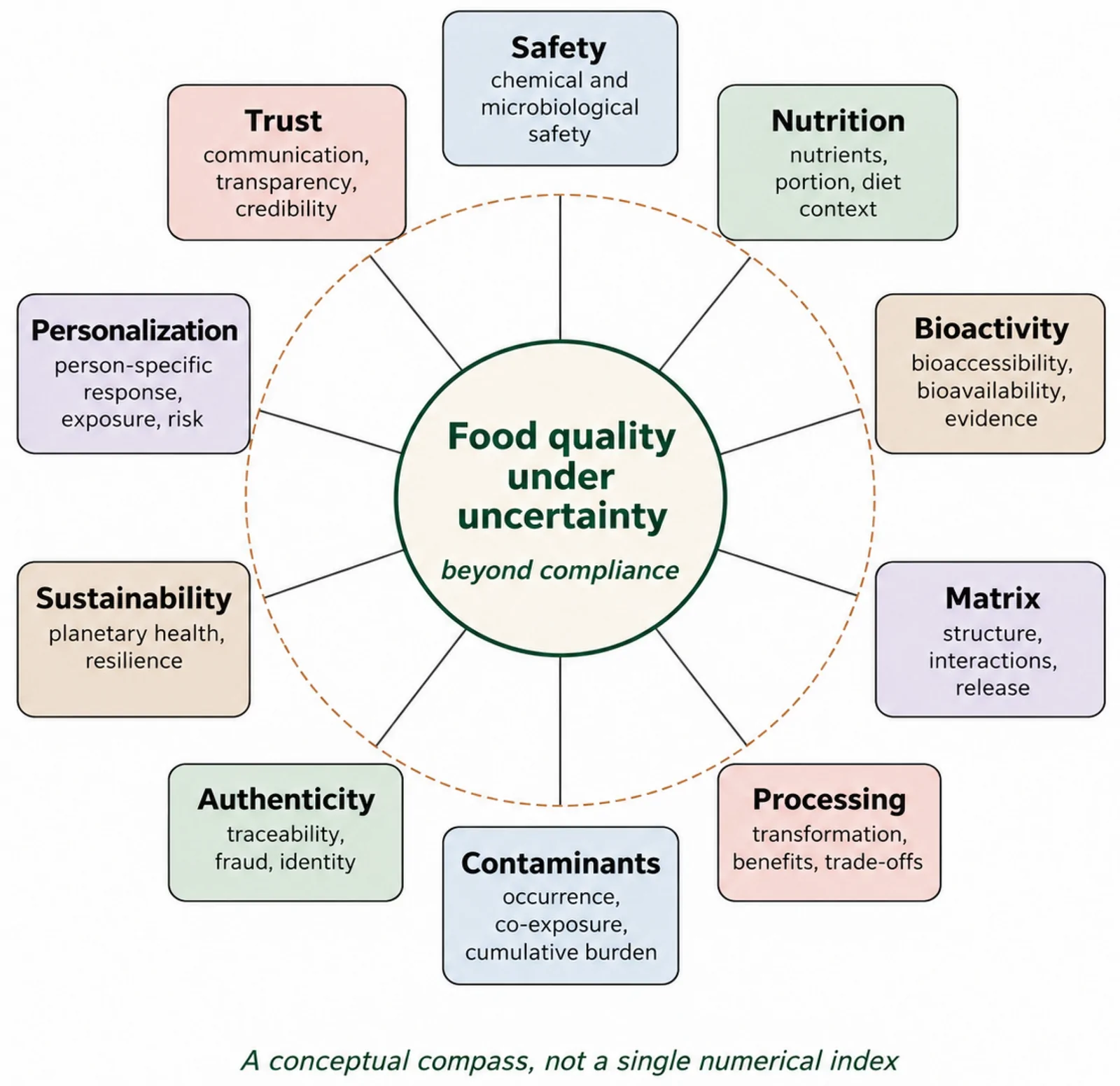
New Food Quality Compass for multidimensional assessment of food quality and safety under uncertainty. The ten dimensions are considered in relation to the product, consumption pattern, exposure, and decision context. The Compass is not a numerical index, and no fixed weighting is assigned to individual dimensions.

The *New Food Quality Compass* presents food quality not as a static property of a product but as a dynamic profile of benefits, risks, uncertainties, and trust. This perspective provides the basis for the subsequent analysis of quality paradoxes across different food categories and of future tools for food quality assessment (Figure 2). 

## 5. Food Categories and Quality Paradoxes: Minimally Processed, Traditional, Organic, Conventional and Ultra-Processed Foods

Divisions into minimally processed, traditional, organic, conventional, and ultra-processed foods are useful descriptive categories [12,17], but they are not independent indicators of quality [60,61,62,63]. In the following analysis, they are treated as different configurations of composition, matrix, technology, safety, exposure, and consumption context.

### 5.1. Minimally Processed Foods: Freshness, Matrix and Microbiological Vulnerability

Minimally processed foods are usually perceived as being closest to the original raw material and are therefore intuitively associated with freshness and limited alteration of naturally occurring nutrients and bioactive compounds [63,64,77]. One of their strengths is the preservation of tissue structure and the food matrix, which may slow the release of components during digestion, increase satiety, and alter the glycemic response compared with highly comminuted or technologically reconstructed products [63,78].

Minimal processing, however, does not mean minimal risk. In fresh and fresh-cut fruits and vegetables, removal of natural protective barriers, cutting, peeling, washing, packaging, and storage may accelerate tissue respiration, enzymatic reactions, loss of sensory quality, and microbial growth [77,79]. Reviews of the safety of fresh plant products emphasize that contamination can occur at many stages, from the field, water, manure, animal contact, and manual harvesting to washing, cooling, distribution, and preparation in the household [79,80].

The paradox of minimally processed foods is that a favorable matrix, freshness, and fewer additives may coexist with a shorter shelf life, greater susceptibility to damage, and the need for careful hygiene management. Quality assessment cannot stop at describing a product as fresh or natural; its susceptibility to change, packaging, storage temperature, cold-chain logistics, and the likelihood of microbiological exposure also need to be considered [77,79,80].

### 5.2. Traditional Foods: Heritage, Identity and the Need for Modern Safety Interpretation

Traditional foods occupy a particular place in the quality debate because they link product characteristics with cultural memory, local origin, production methods, sensory properties, and consumer trust. Trichopoulou et al. noted that traditional products form part of culinary heritage and dietary patterns, while their full characterization is often missing from food-composition databases [81]. Consumer studies, in turn, show that traditionality is understood not only as an “old recipe”, but also in terms of continuity, locality, familiarity, a particular production method, taste, and intergenerational transmission [82].

The paradox of traditional foods lies in the fact that the very features that create their value—locality, artisanal production, variability of raw materials, limited standardization, and cultural proximity—may complicate standardization and the assessment of quality consistency [81,82]. Traditional products may have high sensory and cultural value, but for many of them, particularly fermented products, safety also depends on control of raw materials, process hygiene, fermentation, water activity, acidification, temperature, ripening time, and storage conditions [81,83,84,85].

Fermented foods provide a good example. Historically, fermentation was one of the most important technologies for food preservation, improving sensory properties, and increasing the availability of certain components, while current research also points to the potential relevance of fermented foods for the microbiota and metabolic functions [83,84]. Here too, however, “fermented” should not simply be equated with “health-promoting”. The actual value depends on the microbial species and strains involved, process conditions, product stability, salt and alcohol content, biogenic amines, and the level of evidence supporting the claimed effect [83,84].

### 5.3. Organic Foods: Lower Chemical Input, Consumer Trust and Contextual Limitations

Comparative studies indicate that organic products, on average, are less likely to contain detectable pesticide residues, while differences in phenolic compounds or cadmium have also been observed in some groups of plant foods [86,87,88]. Evidence for a clear nutritional advantage remains heterogeneous, however, and depends on factors such as species, growing conditions, season, and production practices [86,87,88]. Differences between production systems should be interpreted at the level of specific parameters rather than as a general hierarchy of quality [86,87,88,89,90,91].

Another paradox concerns sustainability and availability. Organic agriculture may improve some environmental and social indicators, but its productivity is often lower than that of conventional systems, although the size of this difference depends on crop type, local conditions, and management practices [89,90,91]. Organic food should not be presented as a simple answer to all questions of quality, safety, and sustainability. It is better understood as one possible strategy for shaping food quality, requiring multidimensional assessment across chemical, microbiological, nutritional, environmental, economic, and social dimensions [88,89,90,91].

### 5.4. Conventional Foods: Productivity, Standardization and the Cost of Intensification

Conventional food production is often contrasted with organic production, but this distinction also requires caution. Conventional systems have enabled higher productivity, standardization of raw materials, greater stability of supply, and widespread use of preservation technologies and quality-control procedures [12,40,41,89,90].

Production intensification also carries costs that are not always visible in conventional assessment of the final product. These include pesticide and fertilizer use, pressure on soil and water resources, biodiversity loss, and emissions associated with food production [20,88,89,90,91]. From the perspective of food quality, a conventional product may be safe and compliant with standards while still raising questions about the environmental and social costs of its production [20,89,90,91].

Conventional production should not be treated as synonymous with low quality, just as organic production should not be regarded as a guarantee of the highest quality. Both systems encompass considerable variation in quality profiles. What matters are the specific production practices, productivity, residue management, and the effects of the production system on soil, water, and biodiversity [41,88,89,90,91].

### 5.5. Processed Foods: Preservation, Fortification, Convenience and Matrix Transformation

Processed food is a very broad category and cannot be assessed uniformly. Processing includes relatively simple operations such as cooking, freezing, drying, pasteurization, fermentation, and packaging, as well as more complex industrial processes including extrusion, refining, fractionation, concentration, reconstitution, and texture design [12,13,14]. In many cases, processing is essential for food safety, shelf life, digestibility, waste reduction, transport, and economic accessibility [12,40,62].

The paradox is that the same technology may have effects in opposite directions. Thermal processing can inactivate pathogens and antinutritional factors, but it may also reduce the content of heat-sensitive vitamins or promote the formation of process-derived compounds [12,13,14,92]. Extrusion can improve the functional properties of starches and proteins, enable the production of convenient foods, and increase digestibility, while also altering component structure, the stability of bioactive compounds, and the formation of Maillard reaction products [13,14]. Fortification can help address nutrient deficiencies, but it does not resolve problems related to an unfavorable matrix, high energy density, or rapid eating rate [62,63,93].

Assessment of processed foods should consider not only the degree of processing, but also the purpose and intensity of the process, its effects on the matrix and compositional profile, safety, portion size, and the role of the product in the overall diet. Weaver et al. [62] showed that processed foods can make an important contribution to nutrient intake, whereas Poti et al. [94] reported that greater processing and convenience may be associated with lower nutritional quality of foods purchased by households.

A recent perspective by Schottroff et al. treats food processing not as an isolated technological operation but as a strategic link between raw-material production, distribution, and consumption. Its further development should take account of safety, nutritional value, affordability, food-system resilience, and environmental constraints at the same time [95].

### 5.6. Ultra-Processed Foods: Beyond Nutrient Composition

Ultra-processed foods are extensively studied in relation to diet quality and health outcomes. The NOVA classification shifted attention from nutrient composition alone to the nature, extent, and purpose of processing, drawing attention to industrial products created not simply by preserving raw materials, but through formulations of ingredients, additives, and structures designed for high palatability, convenience, shelf life, and intensive consumption [17,96,97].

Epidemiological data indicate that a high proportion of ultra-processed foods in the diet is generally associated with lower nutritional quality, a higher contribution of added sugars, greater energy density, and a higher frequency of adverse health outcomes [16,96,97,98]. Studies from the NutriNet-Santé cohort have linked greater consumption of ultra-processed foods with higher risks of cancer and cardiovascular disease [99,100], while systematic and umbrella reviews report associations with a broad range of adverse health outcomes, although the quality of the evidence varies [16,98].

The controlled study by Hall et al. provides an important argument in this discussion: an ultra-processed diet increased spontaneous energy intake and body weight despite matching of major nutritional parameters [15]. This finding suggests that the issue of ultra-processed food may extend beyond conventional label information on energy, protein, fat, sugar, and sodium. Texture, eating rate, energy density, the degree of matrix disruption, palatability, portion availability, additives, and eating behavior may also contribute [15,63,93].

The discussion of ultra-processed foods nevertheless requires scientific caution. Some authors have pointed out that definitions of ultra-processed food are not always applied consistently and that the NOVA classification has been criticized for grouping very different products within a broad category [101]. This does not diminish the relevance of ultra-processing as a research question. Rather, future quality assessment should combine processing classification with analysis of the food matrix, energy density, additives, process-derived compounds, portion size, frequency of consumption, and the overall dietary pattern [15,17,93,101].

### 5.7. Synthesis: Food Categories as Multidimensional Quality Profiles

The analysis shows that none of the categories discussed is, by itself, a guarantee of either high or low quality. Their significance depends on composition, matrix, the purpose and intensity of processing, safety, exposure, environmental impact, and consumption patterns. These categories should be regarded as different quality profiles within the *New Food Quality Compass*, rather than as fixed labels of health value or risk (Table 2).

## 6. Chemical Contamination and the Sub-Threshold Multi-Contaminant Burden

Chemical contamination remains one of the central areas of food-quality assessment, yet its interpretation can no longer be limited to the question of whether a single compound exceeds an established limit. Consumers are not exposed to isolated substances, but to diets composed of many products that may contain pesticide residues and other compounds considered in cumulative risk assessment [3,4,5], substances occurring in chemical mixtures [6,7], mycotoxins and process contaminants [21,92], as well as PFAS, micro- and nanoplastics, and substances migrating from packaging [9,10,11,113,114]. At the population level, analytical data need to be linked with actual consumption patterns, including through Total Diet Studies [115,116].

In this context, the proposed concept of *sub-threshold multi-contaminant burden* can be used to describe situations in which individual contaminants remain below legal limits, while a product, a group of products, or the diet as a whole contains multiple chemicals occurring simultaneously at levels below their respective threshold values. This is neither a regulatory term nor evidence of harm, but an interpretive framework that distinguishes product compliance from the long-term exposure profile under real dietary conditions [3,4,117].

A chemical burden understood in this way reveals the limits of the classical compliance model. A legal limit, such as a maximum residue limit (MRL) for a pesticide or a maximum level for a selected contaminant, remains an essential tool for consumer protection, but by itself it does not describe combined exposure arising from many products, many compounds, and repeated consumption over days or years [3,4,118,119,120].

Mixture assessment has developed precisely because dietary exposure is repetitive, complex, and often multi-source. WHO/IPCS proposed a framework for assessing combined exposure to multiple chemicals [117], while EFSA developed a harmonized methodology for combined-exposure assessment [3,121]. Subsequent work indicates that food-related risk assessment is moving beyond simple indicators such as the hazard index toward more operational, component-based, and probabilistic models [122,123,124].

Interpretation of the *sub-threshold multi-contaminant burden* concept requires caution. The presence of multiple trace residues does not automatically indicate a health hazard. What matters is their co-occurrence, the duration of exposure, similarity of mechanisms of action, consumer susceptibility, and uncertainty arising from analytical and toxicological limitations [3,6,7,125,126].

### 6.1. Single-Compound Compliance Versus Cumulative Exposure

Single-compound compliance refers to a situation in which each measured compound is assessed separately against its relevant legal limit, reference value, or health-based guidance value. This model remains the basis of official food control because it is transparent, enforceable, and based on validated analytical methods and formally adopted safety criteria [3,4,118,119,120].

The limitation becomes apparent when analytical results are interpreted only as a set of independent decisions: below the limit or above the limit. A product may comply with all requirements for individual residues while still containing several compounds that affect the same organ, the same physiological system, or similar toxicological pathways [3,118,127]. In such a case, the statement “no exceedances” is legally correct, but it does not fully answer the scientific question of the combined exposure profile [3,4,123,125].

EFSA introduced the concept of cumulative assessment groups, in which substances can be considered together because of a common toxicological effect or target organ [118,127]. Practical application of this approach is illustrated by pilot cumulative-risk assessments for pesticide residues with acute effects on the nervous system and chronic effects on the thyroid [119,120]. These examples are important because they show the transition from a general call to consider mixtures toward operational tools for dietary risk assessment [119,120,128].

Cumulative exposure, in turn, refers to total or combined exposure to multiple compounds, usually weighted according to relative toxicity, a common effect, or contribution to overall risk. Such assessment requires not only analytical data on chemical occurrence in food, but also information on consumption, dietary variation, between-consumer variability, uncertainty, and often probabilistic exposure modeling [4,119,120,128].

From the perspective of food quality, the distinction between compliance and exposure is fundamental: the first concerns whether a product or batch meets legal requirements, whereas the second concerns the burden arising from the actual diet within a defined population and over a given period [3,115,116,124]. Similar questions arise for other contaminant groups, particularly mycotoxins, for which co-occurrence, modified forms, and mixture-assessment models are the subject of separate research [129,130].

**Figure 3:**
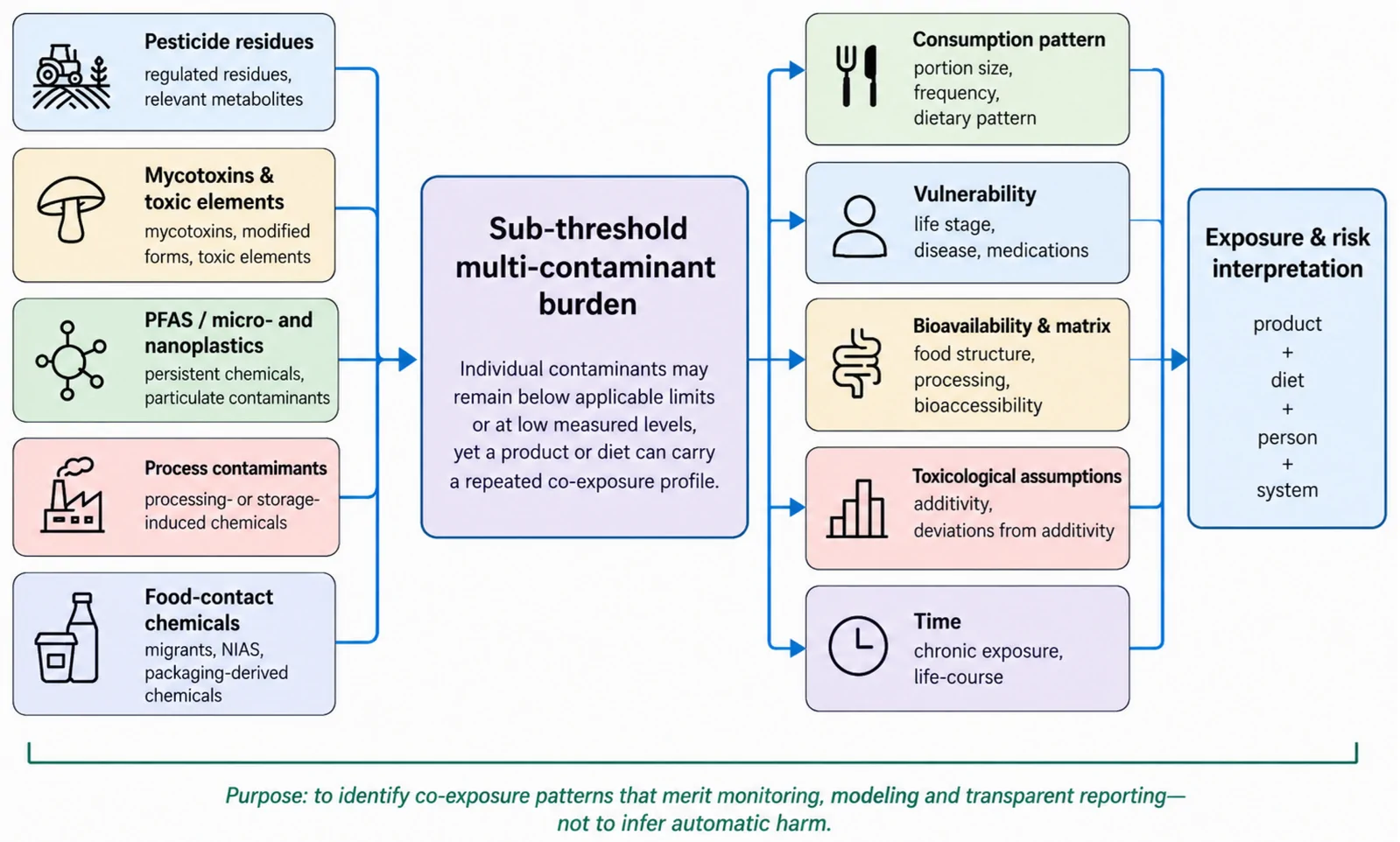
Conceptual model of sub-threshold multi-contaminant burden. Recurrent co-exposure to low levels of multiple contaminants is interpreted in relation to consumption patterns, consumer susceptibility, food matrix and bioavailability, toxicological assumptions, and exposure duration. The model is intended to support screening and prioritization; it does not, by itself, demonstrate an adverse health effect.

The *sub-threshold multi-contaminant burden* model shows that the absence of exceedances for individual compounds does not end the interpretation of exposure, nor does it determine whether further mixture assessment is needed when a product or the overall diet creates a recurrent co-exposure profile involving multiple classes of compounds at low levels (Figure 3). Figure 3 integrates concepts from combined-exposure assessment, cumulative pesticide risk assessment, and the dietary-exposome perspective into the proposed *sub-threshold multi-contaminant burden* framework [3,4,26,124,128].

### 6.2. Low-Dose Chronic Exposure and Mixture Toxicology

Dietary exposure to contaminants is usually chronic and low-dose. Consumers are rarely exposed to a single high dose of one compound; more often, over many years, they consume mixtures of numerous substances present in food at trace or low levels, whose significance depends on toxicity, frequency of occurrence, consumption pattern, age, health status, and individual susceptibility [3,26,117,126].

Additivity, synergy, and antagonism are central concepts in mixture toxicology. Additivity means that the combined effect corresponds to the effect predicted from the summed contributions of individual components; synergy refers to an effect greater than predicted, whereas antagonism refers to an effect smaller than expected [117,131,132]. Systematic reviews of mixture studies emphasize, however, that demonstrating true synergy requires careful experimental design, an appropriate reference model, and cautious interpretation [131,132].

For food risk assessment, dose additivity is particularly useful for groups of compounds that share a similar effect or mechanism of action. This is a protective and operational approach because it allows substances to be grouped into cumulative assessment groups (CAGs), relative potency factors to be applied, or tools such as Monte Carlo Risk Assessment (MCRA) to be used, rather than attempting to test experimentally every possible combination of substances occurring in the diet [118,127,128].

Mixture toxicology cannot, however, be reduced to the assumption that all effects can be predicted by simple dose addition. Real mixtures may contain substances with different mechanisms of action, different metabolic rates, different target organs, and different toxicokinetic and toxicodynamic interactions [117,122,126]. Newer methodologies place greater emphasis on problem formulation, tiered assessment, substance grouping, uncertainty analysis, and selection of tools proportionate to the likelihood of risk [3,117,122,126,127].

In the context of food quality, the mere presence of a mixture should not be equated with an actual health hazard. A more appropriate question is whether there is a biological, toxicological, and exposure-based rationale for considering a given mixture relevant to a particular health effect, consumer group, or consumption scenario [3,117,122,126]. Such cautious interpretation helps avoid both false reassurance and unjustified alarmism [42,75,124].

### 6.3. Pesticides, Mycotoxins, Metals, PFAS, Process Contaminants and Packaging-Derived Chemicals

Pesticide residues are the most developed example of cumulative risk assessment in food. In this area, methodological foundations and regulatory applications have been established for cumulative assessment groups, relative potency, probabilistic exposure assessment, and uncertainty characterization [4,118,119,120,128].

Pesticide residues provide a useful model, but they do not exhaust the problem of chemical food quality. Mycotoxins such as aflatoxins, ochratoxin A, fumonisins, trichothecenes, zearalenone, beauvericin, and enniatins often co-occur in cereals, nuts, spices, dried fruits, and feed, and their profiles may shift with climate, technology, and storage conditions [21,129,130]. For mycotoxins, co-occurrence, masked or modified forms, and the limited amount of data available to predict mixture effects under real dietary conditions are particularly important [21,129,130].

Metals and metalloids such as cadmium, lead, mercury, and inorganic arsenic require assessment primarily in terms of chronic exposure from the whole diet. Their significance depends on concentrations in individual food categories and on consumption patterns, which makes approaches combining monitoring data with Total Diet Studies particularly useful [26,115,116].

PFAS illustrate how contemporary food contaminants can be persistent, global, and derived from multiple sources. EFSA proposed a tolerable weekly intake for the sum of four PFAS, which itself reflects a move away from single-substance assessment toward a group-based approach [113]. More recent relative potency factor methods developed for PFAS further support the need to account quantitatively for mixtures and the relative potency of individual compounds [113,133].

Acrylamide is a well-documented example of a contaminant formed during thermal processing of food [92]. A broader range of process contaminants is discussed in Section 7.

Substances migrating from food-contact materials occupy a distinct place in this discussion. Food-contact chemicals include intentionally used substances, degradation products, and non-intentionally added substances, and their occurrence in food depends on material composition, contact time, temperature, fat content of the food, pH, processing conditions, and storage conditions [8,9]. Micro- and nanoplastics further illustrate how the boundary between packaging, the environment, and the food product is becoming increasingly difficult to define [10,114].

These contaminant groups show that the chemical quality of food is shaped at many stages, including primary production, the environment, processing, packaging, storage, culinary preparation, and international trade. They form a dynamic exposure profile spanning production, processing, packaging, storage, and consumption; classical and emerging contaminants may occur within the same product or diet.

### 6.4. From MRL-Based Control to Exposure-Based Risk Interpretation

The move from MRL-based control to exposure-based risk interpretation does not mean abandoning regulatory limits. Maximum residue levels, maximum contaminant levels, and health-based guidance values remain essential for official control, trade, producer responsibility, and consumer protection [3,4,5]. Their limitation is that a limit for one compound in one product does not automatically answer the question of total dietary exposure [3,4,117,128].

Exposure-based interpretation requires analytical data to be linked with consumption data. Total Diet Studies are particularly important in this regard because they allow long-term dietary exposure to chemical hazards to be estimated more realistically than monitoring of individual raw commodities, as foods are analyzed in forms closer to those actually consumed [115,116]. This approach is especially relevant for contaminants of chronic concern and for populations with different consumption patterns [115,116].

Probabilistic models are playing an increasing role in cumulative risk assessment. They can account for variability in contaminant concentrations, variability in food consumption, differences between population groups, data uncertainty, and correlations between the occurrence of different compounds [4,119,120,128]. Tools such as MCRA can combine occurrence data, consumption, relative potency, and uncertainty to estimate cumulative exposure [128].

Exposure-based interpretation also has implications for communicating results. The statement that a product does not exceed any MRL answers the legal question of whether a batch complies with requirements, but it does not indicate the contribution of that product to the overall diet or the significance of its consumption for a particular group of consumers [3,4,115]. Modern food-quality control should develop two parallel pathways: assessment of product compliance and assessment of dietary burden at the population level and under defined consumption scenarios [3,115,116,119,120].

### 6.5. Remaining Research Gaps: Monitoring, Data Integration and Prioritization

Remaining research gaps concern substances not included in routine monitoring and the integration of analytical, consumption, biomonitoring, and toxicological data. Suspect and non-target screening (NTS) approaches and foodomics can broaden the range of observation, but the resulting signals require prioritization and cautious interpretation [23,56,57,134]. Assessment of *sub-threshold multi-contaminant burden* additionally requires co-occurrence data to be linked with consumption, bioavailability, and uncertainty [26,124,128]. A detailed measurement agenda, including the development and validation of multi-contaminant burden metrics and realistic mixture studies, is presented in Section 15.

## 7. Emerging Contaminants and Hidden Exposures

Emerging contaminants and hidden exposures should be understood not only as entirely new chemicals, but also as hazards that were already present in the food system and have become more visible with the development of high-resolution analytical methods, biomonitoring, mixture toxicology, and databases [8,9,135,136]. In this sense, “emerging” often refers to new knowledge about sources of exposure, migration, persistence, accumulation, and regulatory difficulties rather than simply to the novelty of the compound itself.

The greatest challenge for conventional monitoring is posed by hidden exposures: migrants and NIAS from packaging and processing equipment, pesticide metabolites, degradation products, micro- and nanoplastic particles, process contaminants, and toxins whose occurrence changes under the influence of climate, trade, and new technologies [8,9,10,25,136]. Section 7 focuses on hazard groups for which targeted monitoring needs to be supplemented by suspect screening, non-target screening, exposure assessment, biomonitoring, and toxicological interpretation [22,23,24,25,56].

### 7.1. PFAS: Persistent Chemicals, Dietary Exposure and Global Redistribution

Per- and polyfluoroalkyl substances (PFAS) illustrate the limitations of the conventional approach to assessing the chemical quality of food. Their environmental persistence, resistance to degradation, widespread industrial use, presence in food-contact materials, and potential for bioaccumulation mean that dietary exposure cannot be explained solely by local food-production practices [113,135,137,138].

EFSA has identified PFAS as an important food-safety concern, emphasizing the need to assess combined exposure to selected compounds in this group and establishing a tolerable weekly intake for the sum of PFOA, PFNA, PFHxS, and PFOS [113]. The toxicological and environmental literature also increasingly argues that PFAS should be considered as a class of substances, because single-compound assessment may not keep pace with the very large number of chemical structures, substitutes, and transformation products involved [137,138].

Sources of PFAS in food are diverse. They may arise from contamination of the primary production environment, water, soil, feed, and aquatic organisms, as well as from migration from food-contact materials, particularly where resistance to grease, water, or staining is required [135,137]. The review by Phelps et al. shows that PFAS in food packaging should be considered together with migration, toxicity, substitution of one PFAS by another, and risk management throughout the material life cycle [137].

Qiu et al. showed that international trade in marine fish can redistribute PFAS exposure between regions [11]. Assessment of the quality of fish products should consider not only species, freshness, and nutritional value, but also origin, trophic level, harvesting environment, import status, and supply-chain traceability [11,113,135].

PFAS also provide a useful example of the mixture problem. Even when individual compounds are detected at low levels, consumers may be exposed simultaneously to multiple congeners, with contributions from food, water, food-contact materials, and the domestic environment [113,133,138]. Future assessment will need to link PFAS occurrence data in food with biomonitoring, dietary models, and group-based approaches to risk assessment [113,133,138].

### 7.2. Micro- and Nanoplastics: Particles, Analytical Uncertainty and Food-Contact Pathways

Micro- and nanoplastics are another example of an exposure that does not fit easily within the conventional model of chemical analysis. They are not a homogeneous group of compounds but a collection of particles that differ in polymer type, size, shape, surface properties, additives, degree of aging, and ability to carry other substances [114,139,140]. As early as 2016, EFSA pointed to major knowledge gaps concerning micro- and nanoplastics in food, particularly seafood [114], while more recent studies show that the issue has expanded considerably into food analysis and food-contact materials [139,140].

Zimmermann et al. presented a systematic evidence map showing that food-contact materials may be a source of micro- and nanoplastics in food and food simulants [10]. This is important because discussion of microplastics was for many years centered largely on the marine environment and seafood, whereas food may also be exposed through ordinary contact with packaging, utensils, equipment components, processing, or consumer preparation [10,139].

The main limitation in this field concerns not only particle toxicology but also the quality and comparability of analytical data. Duncan et al. noted that analysis of micro- and nanoplastics in food requires standardization of sampling, prevention of laboratory contamination, sample preparation, polymer identification, particle-size reporting, and quantitative criteria [139]. Canga et al. further emphasized that results from studies of microplastics in foods and beverages are often difficult to compare because of methodological differences, inconsistent reporting, and variable data quality [140].

Early estimates of human microplastic intake, such as the study by Cox et al., drew attention to the possibility of chronic dietary exposure, while also demonstrating how strongly the estimates depend on limited input data and assumptions about consumption [141]. More recent analytical approaches, including pyrolysis-GC-MS, can support quantitative assessment of polymer mass, but they still require careful interpretation because particle-based, spectroscopic, microscopic, and thermochemical methods provide different types of information [139,140,142].

In the context of food quality, micro- and nanoplastics represent an area of dual uncertainty: toxicological and analytical. This does not imply that every product containing trace amounts of particles should automatically be classified as unsafe. It does mean, however, that future quality monitoring should develop comparable methods, blank controls, reporting standards, and exposure models based on realistic consumption scenarios [114,139,140,141,142].

### 7.3. Food-Contact Chemicals and NIAS: The Hidden Chemistry of Packaging and Processing Interfaces

Food-contact materials are an integral part of the food-quality system because they influence shelf life, microbiological safety, ease of distribution, and reduction of food losses [12,40]. They also create a source of hidden chemical exposure, because compounds present in packaging, coatings, adhesives, inks, plastics, paper, cardboard, rubber, metals, or processing equipment can migrate into food [8,9,143].

Geueke et al. compiled evidence of widespread human exposure to food-contact chemicals, showing that many substances associated with food-contact materials can be detected in human biological samples [9]. Muncke et al., in turn, emphasized important limitations of current safety assessment for food-contact chemicals, particularly in relation to mixtures, unidentified substances, migration from multilayer materials, recycling, and the lack of toxicological data for many compounds [8].

A distinct category consists of non-intentionally added substances (NIAS), compounds that are not added for a technological purpose but arise as reaction products, degradation products, raw-material contaminants, products of material aging, component interactions, or recycling processes [25,102,103]. NIAS are more difficult to control than intentionally added substances because they are often absent from material ingredient lists, may occur at low levels, can form complex profiles, and may require high-resolution methods for identification [25,102,103].

The FCCmigex database and the associated publication by Geueke et al. showed that many compounds migrating or extractable from food-contact materials are not included on lists of substances authorized for use [143]. From a *beyond compliance* perspective, formal compliance of a material with requirements for known substances does not exhaust the assessment of possible migration of unidentified compounds, degradation products, or substances formed during processing and use [8,102,103,143]. More recently, systematic prioritization of 15,159 known food-contact chemicals has further illustrated the scale of this chemical space and the need for hazard- and exposure-informed grouping and prioritization strategies [144].

Assessment of NIAS requires analytical chemistry and toxicology to be considered together. The guidance by Nerín et al. shows that selection of analytical methods must take account of volatility, polarity, molecular mass, expected migration level, material type, and the food matrix [102]. More recent reviews of non-target analysis also stress that detection of an HRMS signal alone is insufficient; confidence in identification, toxicological prioritization, exposure assessment, and cautious regulatory interpretation are also required [25,56,103].

### 7.4. Process Contaminants and Mineral Oil Hydrocarbons: Technology-Related Chemical Exposures

Process contaminants illustrate the trade-off between technological benefits and the formation of unwanted reaction products. They arise not because processing is unnecessary, but because many useful operations—heating, frying, baking, roasting, smoking, drying, refining, or fermentation—can simultaneously promote desirable and undesirable chemical transformations [12,92,145].

Acrylamide, furans, 3-MCPD and its esters, glycidyl esters, polycyclic aromatic hydrocarbons, and selected Maillard reaction products show that food quality cannot be assessed solely on the basis of raw-material freshness or declared nutritional composition [92,145,146]. The classic study by Mottram et al. demonstrated a mechanistic link between the Maillard reaction and acrylamide formation, whereas later EFSA assessments and reviews have emphasized the need to combine risk-reduction measures with maintenance of acceptable sensory and technological properties [92,146].

The presence of process contaminants does not automatically indicate a technological failure. Some arise under typical conditions of food preparation in both households and industry. Risk management focuses on reducing their levels, optimizing process parameters, selecting appropriate raw materials, and education rather than on eliminating every chemical reaction involved [92,145].

Hidden exposures also include mineral oil hydrocarbons, comprising mineral oil saturated hydrocarbons (MOSH) and mineral oil aromatic hydrocarbons (MOAH). Their sources may be associated with mineral oils, lubricants, packaging materials, recycled paper and cardboard, processing aids, environmental contamination, or contact during production and transport [147,148]. EFSA updated its risk assessment of mineral oil hydrocarbons in food, indicating that this group requires particular attention because of the complexity of the mixtures, analytical difficulties, and toxicological differences between MOSH and MOAH [148].

### 7.5. Pesticide Metabolites and Transformation Products: Beyond Parent Compounds

Assessment of pesticide residues has traditionally focused mainly on parent compounds, for which maximum residue levels are established and routine monitoring is performed. Metabolites and transformation products are becoming increasingly important, however, because they may form in plants, soil, water, animals, during storage, or during food processing [4,149].

Pelkonen et al. showed that metabolites are an important component of regulatory pesticide risk assessment because the toxicity, persistence, and exposure relevance of transformation products do not always correspond to the properties of the parent compound [149]. A comprehensive assessment of the chemical quality of food should consider not only lists of active substances but also profiles of metabolites, degradates, and reaction products formed under actual production and processing conditions [4,149].

This issue is particularly relevant to non-target and suspect screening. If an analytical method is designed only for known active substances, some transformation products may remain undetected or uninterpreted [23,24,56,57]. Future residue monitoring should combine regulatory approaches with methods capable of capturing unexpected signals, followed by prioritization according to toxicological and exposure relevance [23,24,56,57,149].

### 7.6. Climate-Sensitive Toxins and Changing Exposure Patterns

Some hazards described as emerging are not chemically new; what is emerging is their changing pattern of occurrence. Climate change may affect temperature, humidity, plant stress, and the geographical distribution of pathogens and pests, thereby altering the risk of mycotoxins and other natural toxins in food and feed [21,136].

Casu et al. noted that the influence of climate on mycotoxin contamination is becoming an increasingly important food-safety issue because changing environmental conditions can shift geographical and seasonal risk profiles [21]. Quality-control systems should not rely solely on historical hazard maps, but should also incorporate predictive monitoring, modeling, meteorological data, and seasonal and regional scenarios of toxin occurrence [21,136].

Other hazards influenced by the environment and the supply chain may be considered in the same context, including algal toxins, plant alkaloids, contamination associated with drought or flooding, changes in storage practices, and pressure from emerging pathogens [136]. Not all of these hazards are new, but their importance may increase as a result of climate change, shifts in raw-material sources, international trade, and relocation of production to new regions [21,136].

### 7.7. From Hidden Exposures to Early Warning: Analytical and Regulatory Implications

For monitoring, the main implication is the need to combine targeted analysis with screening tools and early-warning systems, particularly when routine analyte lists do not include new or poorly characterized exposures [23,37,136]. Signal identification, toxicological prioritization, and linkage with exposure data remain essential [25,56,57]. The principal monitoring areas are summarized in Table 3, while the analytical tools are discussed in greater detail in Section 8.

## 8. Foodomics, Exposomics and Analytical Transformation of Food Quality Control

This Section translates the *beyond compliance* concept into analytical practice in food control, moving from targeted methods through *suspect* and *non-target screening* to multidimensional profiling and the linkage of product composition with exposure. Foodomics integrates advanced analytical techniques, omics approaches, and bioinformatics, enabling the assessment of complex profiles of composition, safety, authenticity, and technological change [154,155,156,157]. Interpretation of such data requires multivariate methods and pattern recognition [158], while metabolomics is one of the most developed applications of this strategy in food science [159].

A practical control model is layered. HRMS-based *suspect* and *non-target screening* extend analytical coverage beyond a predefined list of analytes [134,160]. Rapid spectroscopic and imaging techniques can serve as screening tools [161,162,163,164], whereas chemometrics and AI support classification and interpretation of multidimensional data [165,166,167]. Linking these layers with actual human exposure leads to the perspective of *food exposomics* [168]. 

### 8.1. Targeted Monitoring: Strengths and Blind Spots

Targeted monitoring remains one of the main tools of official chemical food control because it enables validated, quantitative determination of predefined analytes and direct comparison of results with established decision criteria. Its strengths lie in selectivity, reproducibility, and the ability to generate results suitable for routine control and confirmation of compliance [154,155,157,169,170].

The main limitation of targeted monitoring is its scope: the method answers questions about compounds that the laboratory deliberately searches for, but it does not automatically capture signals from compounds outside the predefined analyte list [23,24,25,134,160]. Within the *beyond compliance* model, targeted analysis remains the reference and confirmatory layer, complemented by exploratory approaches.

### 8.2. Suspect and Non-Target Screening in Food Safety

The difference between *suspect screening* and *non-target screening* lies mainly in the degree of prior knowledge about the compounds being sought. *Suspect screening* uses lists of substances predicted from databases, process information, food-contact materials, or previous reports, whereas *non-target screening* (NTS) aims at the broadest possible acquisition of signals followed by their annotation and identification [23,24,25,134]. Their common value lies in revealing signals that are not covered by routine targeted analysis and that subsequently require prioritization and, where necessary, confirmation [57,134,160]. 

The main challenge is not generating a large number of signals, but identifying and prioritizing them reliably. HRMS data may include signals from genuine analytes, but also from fragments, adducts, isotopologues, artefacts, and background or matrix components [57,134]. Identification in NTS should be reported together with a confidence level, ranging from accurate mass and molecular formula information through a probable structure to identification confirmed with a reference standard [56]. A *non-target discovery* result is often only the starting point for further analysis, and substances of toxicological or regulatory relevance require subsequent confirmation by a targeted method and, where needed, quantitative determination [23,57,134]. 

Authenticity assessment is another important application of non-target methods. Rather than searching for a single previously known marker, a multidimensional product fingerprint can be analyzed and compared with a reference profile for species, origin, or production method. The use of such models, particularly in official control, depends on appropriate validation, a representative reference set, and transparent reporting [171,172,173]. 

These approaches are also beginning to be used for systematic identification of emerging hazards. The EFSA SCREENER project provides an example of screening tools applied to emerging chemical risks in the food chain [174]. Broader experience with workflow harmonization, data quality, and NTS reporting can also be drawn from guidance developed for environmental monitoring [175], although such documents should be treated as sources of methodological principles rather than as direct guidance for official food control.

### 8.3. Complementary High-Resolution, Elemental, Spectroscopic and Imaging Platforms

No single analytical platform is capable of describing the full chemical space of food. Differences in polarity, volatility, molecular mass, concentration, and matrix properties require complementary techniques selected according to the analytical question and the expected level of information [154,155,156,157].

LC-HRMS (liquid chromatography-high-resolution mass spectrometry) is a key platform for *suspect* and *non-target screening* of non-volatile, polar, and moderately polar compounds, providing accurate-mass and fragmentation data for subsequent signal annotation [23,57,134]. GC-HRMS (gas chromatography-high-resolution mass spectrometry) offers a complementary approach for volatile and semi-volatile compounds and for broad chemical profiling of products [134,154,157].

Complementary information is provided by techniques that cover other parts of chemical space. ICP-MS (inductively coupled plasma mass spectrometry) enables sensitive assessment of elemental profiles [176], whereas NMR (nuclear magnetic resonance spectroscopy) is particularly useful for reproducible fingerprinting and metabolite profiling, although its sensitivity for trace constituents is generally lower than that of MS-based methods [154,157,159]. 

Spectroscopic and imaging techniques shift part of quality control toward rapid, non-invasive screening. FTIR (Fourier-transform infrared spectroscopy), Raman, and hyperspectral imaging can provide fingerprints useful for classification, assessment of heterogeneity, and detection of quality deviations [161,162,167], while SERS (surface-enhanced Raman spectroscopy) expands the possibilities for sensitive detection of selected markers and contaminants [163,164]. Routine use of these approaches still requires robust calibration models, validation, and control of transferability across batches and instruments [161,162].

### 8.4. Chemometrics and Artificial Intelligence in Pattern Recognition

As data become increasingly multidimensional, the challenge in food analysis is no longer limited to obtaining a signal but also includes its interpretation. Chemometrics makes it possible to transform complex instrumental profiles into information on sample similarity, variability, authenticity, or product classification [158,165]. Unsupervised methods such as principal component analysis (PCA) are used mainly to explore data structure, whereas supervised models can support classification and prediction, provided that they are properly validated and overfitting is controlled [158,165,173].

Artificial intelligence and machine learning can assist with signal prioritization, classification of spectra and images, integration of multiplatform data, and recognition of unknown contaminants. A direct example is the use of LC-HRMS and machine learning for screening unknown chemical compounds in food [160], while more recent reviews and reports point to increasing integration of AI into food analysis [166,167,177].

Model reliability, however, still depends on data quality and representativeness, external validation, and robustness to changes in matrix, batch, instrumentation, and laboratory conditions. Control of overfitting and bias, model transparency, auditability, and continued expert oversight are particularly important [165,166,167,177]. Within the *beyond compliance* model, AI should be treated as a tool that supports interpretation and prioritization rather than as an autonomous substitute for chemical, toxicological, or regulatory assessment [165,166,167,177].

### 8.5. Food Exposomics: Linking Food Composition with Real Human Exposure

Food exposomics shifts the unit of interpretation from the product to actual human exposure by linking the chemical profile of food with the amount and frequency of consumption, bioavailability, metabolism, exposure biomarkers, and biological response [26,27,28,29,168]. It does not replace product analysis, but gives it an exposure context: the concentration of a compound in food acquires full exposure relevance only when considered together with the actual pattern of consumption.

A recent perspective proposed a precision-toxicology framework that similarly emphasizes the transition from external occurrence data toward biologically effective and individually variable exposure by integrating food-contaminant data with biomonitoring, mechanistic evidence, and mixture-risk assessment [178].

Food exposomics requires information from several levels that are often considered separately in conventional food monitoring. A concentration measured in food captures only part of external exposure. Interpretation also depends on how much and how often the food is consumed, on bioavailability and metabolism, and, where suitable biomarkers exist, on internal dose and biological response. Time is another important factor. A single food measurement or biomarker result may not adequately represent chronic exposure or within-person variability. Biomarkers also need to be interpreted cautiously when the same compound may originate from dietary and non-dietary sources. Food exposomics is therefore most informative when occurrence data are considered together with consumption, biomonitoring, and, where relevant, mechanistic evidence [27,28,29,168,178].

For food quality and safety assessment, the main value of this approach is not that it replaces targeted analysis or conventional exposure assessment. Instead, it connects product characterization with biologically relevant human exposure. This becomes particularly useful when an analytically compliant product may still require broader interpretation because of co-exposure, an unusual consumption pattern, or greater susceptibility in a particular consumer group [26,29,168,178].

The main limitation remains the integration of data derived from different sources and measurement scales. Further development of food exposomics requires comparable analytical data, validated biomarkers, reliable consumption data, interoperable databases, transparent modeling of uncertainty, and stronger links between compositional information and exposure assessment [26,27,28,29,168]. Actual exposure simultaneously includes contaminants, bioactive compounds, additives, substances migrating from food-contact materials, and their metabolites, so integration of these data requires common standards of quality and interpretation [26,168].

**Figure 4:**
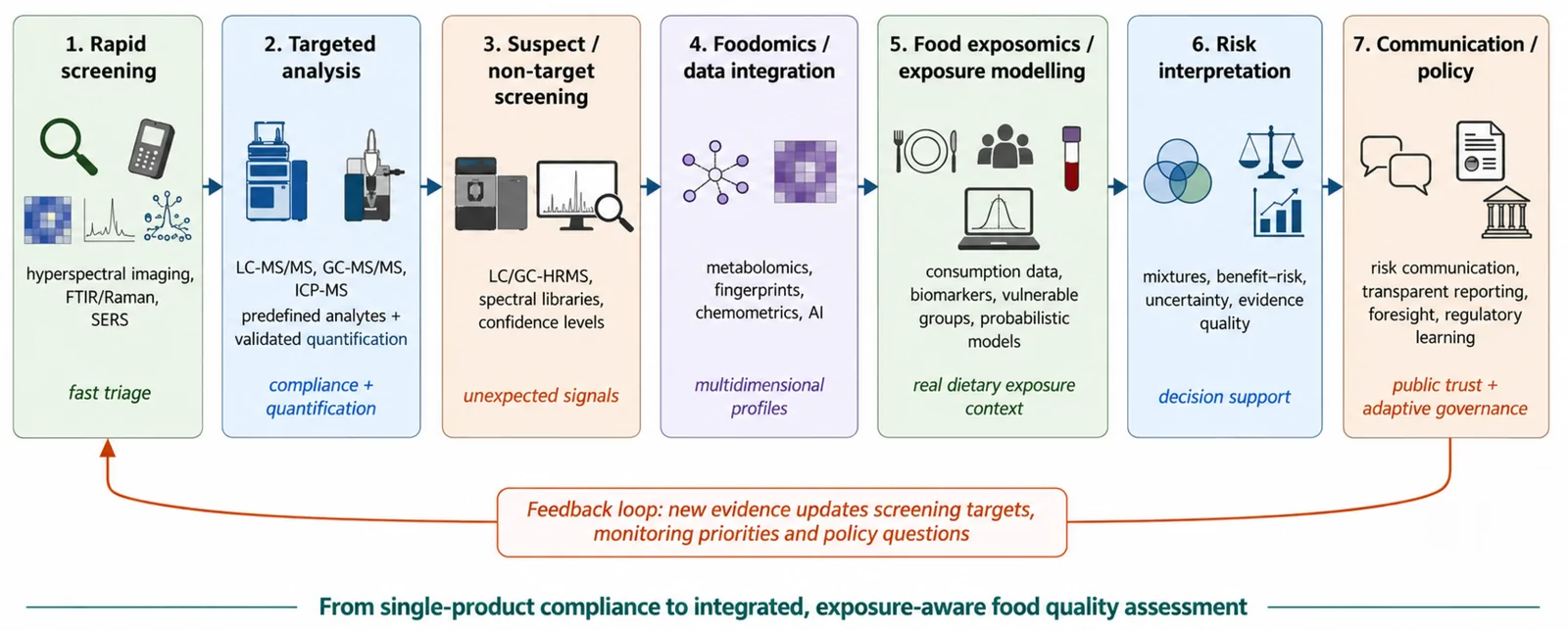
Multilevel analytical-to-decision workflow for food quality and safety assessment. Rapid screening, targeted analysis, suspect and non-target screening, foodomics, exposure modeling, risk interpretation, and communication and policy represent complementary analytical and interpretive layers linked by feedback.

Figure 4 synthesizes the transition from complementary analytical layers—rapid screening, targeted analysis, and suspect/non-target screening—to the integration of multidimensional data [134,154,162]. Linking these data with exposure assessment, further interpretation, and foresight creates a broader analytical-to-decision workflow [37,168]. Table 4 summarizes the functions, strengths, and limitations of the principal analytical tools. 

## 9. Sample Preparation as a Critical Determinant of Analytical Reliability

The analytical tools discussed in Section 8 are useful only when the preceding stages of the workflow provide representative and reliable data. The final result therefore depends not only on instrumental performance, but also on how samples are collected and prepared. Errors related to sampling and sample representativeness [179,180], as well as extraction efficiency and matrix effects [181,182,183], can compromise result quality regardless of the sensitivity and selectivity of the instrumental platform used.

Sample preparation is not merely a technical step, but one of the main filters of analytical information: it determines which part of the actual chemical profile of a product is preserved, extracted, enriched, or lost. Within the *beyond compliance* model, particular importance is attached to representativeness, analyte stability, extraction selectivity, matrix effects, coverage of multiple compound classes, and the suitability of a method for its intended purpose.

### 9.1. Sampling and Representativeness as Sources of Analytical Uncertainty

The first source of uncertainty is sample representativeness. Food analysis usually examines only a fraction of a batch, raw material, or product that is intended to represent a much larger sampling target. If the selected material does not reflect its actual heterogeneity, even a highly precise instrumental result may still be substantively unrepresentative [179,180]. This is particularly important for components and contaminants that are unevenly distributed within a batch or product.

The result should be related to a clearly defined sampling target, and the method description should include at least the sampling strategy, number of primary units, preparation of the composite sample, mass of the laboratory sample, and transport and storage conditions [179,180]. Representativeness is thus a prerequisite for interpreting a result, not merely a technical step preceding analysis.

### 9.2. Homogenization, Stabilization and Storage: Invisible Sources of Bias

Once a sample has been collected, its physical homogenization and stabilization become further sources of uncertainty. Comminution, mixing, drying, freeze-drying, or selection of the analytical test portion should produce material that is as homogeneous as possible without substantially altering the compounds being measured. In multiphase matrices or products in which the analyte is unevenly distributed, inadequate homogenization increases between-subsample variability and may systematically alter the result [180]. If the sample is not analyzed immediately, stabilization and storage conditions should be clearly defined and reported [180].

Another filter of analytical information is the way analytes are released from the matrix. Conventional and energy-assisted extraction techniques differ in how temperature, pressure, and energy affect recovery and component stability [184,185,186,187]. The development of microextraction further shows that miniaturization changes not only solvent consumption but also process selectivity; LPME represents a broad family of such approaches [188], whereas DLLME was originally introduced for extraction from aqueous samples [189].

### 9.3. Matrix Effects, Recovery and Calibration in Quantitative Food Analysis

Matrix effects are one of the main reasons why methods developed in simple standard solutions cannot be transferred directly to complex food matrices. In LC-MS/MS, co-extracted components may suppress or enhance ionization, producing differences between the true analyte content and the observed instrumental response [181,182,183]. Distinguishing between *extraction recovery*, *matrix effect*, and *apparent recovery* is useful, because deviation from the expected value may result either from incomplete extraction or from a matrix-induced change in instrumental response [181,182].

Strategies for reducing matrix effects may include more selective cleanup, dilution of the extract, *matrix-matched calibration*, *standard addition*, or the use of stable isotope-labeled internal standards [181,182,183]. None of these approaches is universal: intensive cleanup may reduce recovery, dilution may impair sensitivity, and isotopically labeled standards increase cost and are not always available. Assessment and correction of matrix effects should form part of validation tailored to the specific matrix and analytical purpose [181,182,183].

### 9.4. QuEChERS and Multi-Class Workflows: Strength, Flexibility and Boundaries

QuEChERS is one of the most influential approaches to multiresidue sample preparation in food analysis. The combination of acetonitrile extraction, salting-out partitioning, and dispersive solid-phase extraction (SPE) provided a rapid and flexible workflow that was subsequently developed through buffered variants, different salt mixtures, and modifications of the cleanup step [53,190,191,192,193].

The development of *multi-class extraction* extended this logic beyond conventional pesticide analysis. Mol et al. demonstrated a generic extraction approach covering pesticides, mycotoxins, plant toxins, and veterinary drugs simultaneously [194]. In parallel, miniaturization and micro-QuEChERS reduce the use of sample material, solvents, and consumables, placing sample preparation within the broader strategy of green sample preparation [55,195,196,197].

Broad analyte coverage, however, always involves compromise. QuEChERS is not universal across all matrices and chemical classes, and the larger the chemical space one extraction is expected to cover, the more difficult it becomes to maintain optimal recoveries, selectivity, and matrix-effect control for every compound [54,193,194,198]. Within the *beyond compliance* model, the value of a method lies not simply in the number of analytes detected, but in a clearly defined account of what the method can—and cannot—reliably cover.

### 9.5. Microextraction and Sorbent-Based Approaches: Selectivity, Enrichment and Miniaturization

Microextraction and sorbent-based techniques reduce sample and solvent consumption while potentially combining extraction, selective enrichment, and sample introduction into the analytical system. Their importance in food analysis stems mainly from the possibility of tailoring selectivity to particular compound classes and from their potential for miniaturization and workflow integration [188,198,199,200,201].

SPME is a particularly mature example of this approach and can combine sampling, extraction, enrichment, and desorption without conventional solvent extraction [199,200]. Sorbent selectivity, temperature, equilibration time, and matrix composition strongly influence the resulting profile, so the high selectivity of microextraction may be both an advantage and a source of bias [200,201]. Miniaturization is valuable only when the chemical scope and limitations of the method are explicitly defined. 

### 9.6. Energy-Assisted and Green Extraction: Balancing Efficiency and Analytical Performance

Energy- and pressure-assisted techniques, such as ultrasound-assisted extraction, microwave-assisted extraction, pressurized liquid extraction, and supercritical fluid extraction, can shorten sample-preparation time, enhance mass transfer, and reduce solvent consumption [184,185,186,187,201]. Greater process intensity, however, does not necessarily improve analytical reliability: temperature, pressure, ultrasound energy, and extraction time may affect compound stability and selectively increase the recovery of only certain matrix components [184,185,186,187].

“Green” should not be treated as synonymous with “better”. Assessment of solvent, energy, reagent, and waste consumption should be combined with validation, information quality, and method functionality. Tools such as AGREEprep and other greenness metrics, together with the concept of White Analytical Chemistry, formalize this balance between environmental impact, analytical performance, and practical utility [196,197,202,203,204]. 

### 9.7. Automation and High-Throughput Sample Preparation

Automation of sample preparation can increase throughput, reduce operator-related variability, improve control over the timing of individual steps, and facilitate integration of extraction and cleanup with the detection system. In food analysis, this includes automated SPE, microfluidic solutions, robotic systems, and direct coupling of sample preparation with instrumental analysis [205]. Its greatest value is seen in well-validated and reproducible workflows, but automation does not eliminate existing sources of error and may systematically reproduce bias introduced during earlier stages of sample preparation [179,180,205]. 

### 9.8. Fit-for-Purpose Sample Preparation for Food Quality Under Uncertainty

There is no single optimal sample-preparation strategy for all food analysis. A method should be *fit-for-purpose*, meaning that it is matched to the intended application, matrix, analyte range, concentration levels, required sensitivity, and depth of validation [169,198,204]. This principle is especially important when results are interpreted beyond the scope of routine analysis, because a method that is reliable for a particular class of compounds does not automatically become a method capable of describing the entire chemical space of the sample.

A practical assessment of sample preparation should consider sample representativeness, analyte stability, extraction range and selectivity, the effect of cleanup, matrix effects, calibration strategy, the validated scope of the method, and reporting quality. This is particularly important in *suspect* analysis, NTS, and foodomics, where sample preparation determines which part of the product’s chemical space remains analytically visible [23,57,134]. These elements form the operational checklist presented in Table 5.

## 10. Future Foods: Opportunities, Constraints and Emerging Uncertainties

The limitations of analytical coverage and sample preparation discussed in Section 8 and Section 9 become especially relevant for *future foods* because new raw materials, matrices, and production processes may change both product composition and the profile of potential hazards. *Future foods* are not a single product category. They represent a diverse group of strategies developed in response to environmental, resource, and food-system pressures [32,33,34,35,36]. They include alternative protein sources, such as plant-based analogues of animal products [206,207,208,209,210], insects, algae, single-cell protein, and precision fermentation [211,212,213,214,215], cultured meat and, more broadly, *cellular agriculture* [216,217,218,219,220], controlled cultivation systems [221,222], underutilized plant species [223] and 3D food printing [224,225]. What these approaches have in common is not technological novelty itself, but the attempt to improve food-system resilience, resource-use efficiency, or the ability to design specific product properties.

*Future foods* should not, however, be presented as automatically safer, healthier, or more sustainable than conventional foods. Each technology creates its own profile of benefits, limitations, and uncertainties [32,33,34]. Its assessment should consider, above all, product composition and matrix, the production process, safety, expected consumption, scalability, and acceptance [33,34,111,112].

### 10.1. Future Foods Between Food Security, Sustainability and Technological Dependence

The development of future foods responds to pressures on resources, climate, and the resilience of food systems [18,19,20,34]. FAO foresight documents, however, indicate that new food sources and production systems require parallel identification of potential hazards and regulatory gaps [34,37].

Greater efficiency, process control, or system resilience may also increase dependence on energy, technical infrastructure, specialized inputs, or the scalability of the technology [208,215,217,219,222]. The quality of *future foods* depends not only on the source material, but on the entire production system.

### 10.2. Plant-Based Alternatives: Matrix Engineering, Nutrition and Processing Paradox

Plant-based alternatives to animal products are designed foods that require the selection of proteins, fats, hydrocolloids, and sensory ingredients, together with processes capable of reproducing particular textural and functional properties [207,208]. Their quality arises from the design of the whole matrix rather than from the simple replacement of one ingredient by another [208].

The paradox of plant-based analogues is that they may support a transition toward reduced dependence on animal-derived foods [207], while many are also characterized by complex formulations and a high degree of processing. Their nutritional quality depends on the source and quality of protein, type of fat, sodium content, fortification, and the overall formulation of the product [208,210]. The label *plant-based* is not, by itself, a sufficient quality category.

Processes such as high-moisture extrusion, heating, and protein fractionation can alter matrix structure and component properties, so assessment of these analogues should take account of formulation, matrix, and nutritional value [13,14,208,210]. 

### 10.3. Edible Insects, Algae and Microorganisms: Protein Diversification and Safety Barriers

Edible insects are often proposed as an alternative source of protein, fat, chitin, and micronutrients, and their potential is associated with efficient feed conversion, limited land requirements, and diversification of food sources [209]. Entomophagy still faces cultural acceptance barriers in many Western countries, while the safety of insect-based products requires control of feeding substrates, microbiota, allergenicity, metals, chemical residues, lipid stability, and processing conditions [209,211]. EFSA opinions on insects as novel foods show that market authorization must be based on detailed characterization of the species, product, process, composition, and anticipated consumption [211].

Algae and microalgae are potential sources of protein and functional components and may reduce direct competition for conventional agricultural land, although broader use is still constrained by difficulties in efficient protein extraction and purification [212].

Microorganisms and *single-cell protein* represent a third route for protein diversification. Yeasts, fungi, bacteria, and microalgae can provide biomass rich in protein and other nutrients. Product quality nevertheless depends on the production organism, substrate, cultivation conditions, biomass composition, and the subsequent steps of separation, stabilization, and further processing [213,214].

### 10.4. Precision Fermentation: Process-Derived Components and Compositional Control

Precision fermentation uses microorganisms as controlled production systems for specific ingredients, including recombinant milk and egg proteins that can subsequently be used in the design of alternative food products [215].

For precision fermentation, the identity and safety of the production microorganism, substrate composition, metabolic by-products, purification efficiency, stability of the final ingredient, and process reproducibility during scale-up become central quality considerations [215]. The technology may reduce some dependence on animal production, but it also shifts quality control toward the quality and stability of the entire bioprocess.

For products falling within *novel food* frameworks, regulatory assessment requires appropriate characterization of the product, the production process, and expected consumption [111].

### 10.5. Cultured Meat and Cellular Agriculture: Promise, Scale-Up and Safety Uncertainties

Cultured meat and, more broadly, *cellular agriculture* are being developed as potential alternatives to part of conventional meat production, although the scale of their actual environmental and economic benefits remains dependent on the technology and production scale [217,219,226]. The gap between technological promise and routine, economically accessible production remains substantial. Early studies outlined the basic biological and technological challenges of producing meat from animal cells, while subsequent analyses highlighted barriers related to bioreactor scale-up, cost, culture media, process sterility, cell differentiation, tissue structure, and consumer acceptance [216,217,219,220].

From the standpoint of quality and safety, cultured meat should be assessed simultaneously as a biological and technological product. This requires control of cell lines, scaffold materials, culture media, growth factors, metabolites, process residues, lipid stability, amino-acid composition, texture, sterility, and possible changes in nutritional profile [112,217]. FAO and WHO have indicated that the safety of cell-based foods requires identification of potential hazards at successive stages of the process, from cell sourcing and proliferation through differentiation, biomass harvesting, processing, packaging, and distribution [112].

Cultured meat should not be assumed to have a lower environmental impact. Life-cycle assessments depend strongly on assumptions about energy sources, media composition, process efficiency, production scale, and the type of meat used for comparison [226,227]. This technology should be regarded as having substantial developmental potential, while still requiring critical assessment of scalability, cost, safety, and actual environmental performance [226,227].

### 10.6. Controlled-Environment Agriculture, Hydroponics and Vertical Farming

Controlled cultivation systems, hydroponics, aeroponics, plant factories, and vertical farming alter the way quality of plant raw materials can be understood. Their advantage lies in the ability to control light, temperature, humidity, nutrient-solution composition, carbon dioxide concentration, hygiene, and harvest time, which may improve yield predictability, reduce dependence on weather conditions, and shorten supply chains. In principle, such systems may enable production closer to consumers, lower water use, reduced pesticide input, and more stable quality parameters [221,222].

Greater environmental control, however, also means greater dependence on energy, technical infrastructure, and continuous monitoring of production parameters. Product quality depends not only on soil-free cultivation or production in an enclosed environment, but also on water and nutrient-solution quality, hygiene, and the stability of the entire production system [221,222].

### 10.7. Underutilized Crops and Neglected Biodiversity as Low-Tech Future Foods

Not all *future foods* need to be high-technology products. Underutilized and neglected plant species may increase production diversity and strengthen food-system resilience, particularly under resource and climate constraints [223]. Their wider adoption, however, requires assessment of composition, nutritional value, technological properties, and production scalability. The future of food includes both advanced bioprocesses and more effective use of existing biodiversity.

### 10.8. Technologically Designed Foods, 3D Printing and Personalized Matrices

Technologically designed foods include products whose properties are intentionally constructed at the level of composition, structure, texture, portion size, release rate of components, stability, or suitability for the needs of selected consumer groups. 3D printing makes it possible to control geometry, texture, multilayer structure, and the spatial distribution of ingredients within a designed product [224,225]. This technology may be particularly useful where modified texture, precise portioning, or personalized composition is required [224,225].

Materials intended for printing must combine suitable rheological properties with the required stability and product quality. The growing number of formulations and process parameters may complicate standardization, reproducibility control, and process validation [224,225].

### 10.9. From Novelty to Evidence-Based Quality Assessment

Assessment of *future foods* should move beyond the technological label toward evidence relating to the specific product and production process. Regardless of category, the main considerations are characterization of composition and matrix, safety, stability, expected consumption, scalability, environmental impact, and consumer acceptance [32,33,34,111]. Within the *New Food Quality Compass*, this means applying the same quality dimensions used for other food categories, but with greater emphasis on process uncertainty, emerging hazards, and the consequences of scale-up.

The central shift is from controlling the finished product to designing quality and safety *ex ante*, in parallel with technological development [34,37]. Food intended for extreme environments provides a particular example of this approach, since requirements for safety, stability, resources, and user needs must be integrated from the design stage onward.

## 11. Space Food as an Extreme-Case Model of Future Food Quality Requirements

Space food represents an extreme model of future food quality because it must simultaneously meet requirements for safety, nutritional value, stability, and acceptability under severe constraints of mass, volume, water, energy, crew time, and possibilities for resupply [35,36,228,229]. A defining feature is the need to maintain this quality over long periods in an environment where intervention options are limited.

Research on space food systems highlights several particularly important areas of uncertainty: long-term nutrient stability [230,231], sensory acceptability and actual food choices made by astronauts [232,233], the microbiological safety of in situ food production [234], and the growing need for more individualized interpretation of nutrition during long-duration missions [235]. This makes space food a useful test case for the concept of *food quality under uncertainty*.

Many of these constraints are not unique to space food. Military operational rations and foods intended for emergency or disaster-relief settings must also provide adequate nutrition and microbiological safety while remaining stable during storage, resistant to packaging failure, compact, easy to prepare, and acceptable to consumers [236,237]. Studies of military rations further show that nutritional and sensory stability during storage depend on preservation technology, packaging, and storage temperature [237]. Space food differs mainly in the extent to which these constraints occur together: storage periods may be much longer, resupply opportunities are extremely limited, food is consumed in a closed habitat, and spaceflight introduces additional physiological demands. Future missions may also require some food to be produced in situ [35,36,228,229]. For these reasons, space food provides an extreme example of how safety, stability, logistics, acceptability, and food-system resilience interact.

### 11.1. Nutrient Stability, Shelf Life and the Problem of Time-Dependent Quality

One of the main constraints of space food is nutrient stability during prolonged storage. Zwart et al. showed that selected components of foods and supplements exposed to long-term conditions aboard the International Space Station (ISS) undergo time-dependent changes, affecting, among others, some vitamins that are sensitive to storage conditions [230]. Cooper et al. analyzed 109 items from the space food system stored for three years at room temperature and reported that some nutrients, including potassium, calcium, vitamin D, and vitamin K, could already be insufficient in relation to nutritional requirements before storage, while declines in vitamins A, C, B1, and B6 were observed during storage [231].

The implications of these studies extend beyond spaceflight. They show that food quality cannot be assessed solely on the basis of composition declared at the time of production. For products intended for long-term storage, what matters is not only their initial quality but also its trajectory over time: nutrient stability and the ability of the product to retain its intended nutritional function [230,231]. 

Long-term stability is closely linked to the product matrix, preservation method, and packaging properties. In a space food system, packaging must simultaneously limit exposure to oxygen, moisture, and light, remain lightweight, and retain functionality under mission conditions [228,229]. Packaging is an integral part of maintaining quality over time rather than merely a unit for distribution.

### 11.2. Microbiological Safety and Process Control in a Closed Habitat

In the closed environment of a space mission, microbiological hazards may have both health and operational consequences. The current system relies mainly on shelf-stable and extensively preserved foods, whereas the move toward in situ production shifts attention from the safety of the finished product to the safety of the entire production process [228,229,234].

Studies of red romaine lettuce grown in the Veggie system aboard the ISS demonstrated that edible material could be produced without detection of the pathogens tested, while also revealing differences between space-grown and ground-control samples in microbial profiles and selected compositional parameters [238]. These findings show that fresh-food production in situ requires simultaneous monitoring of both the product and the cultivation environment.

Under space-production conditions, HACCP principles adapted to an extraterrestrial environment become especially important. Cho and Rhee argue that food production in space requires a tailored system of hazard analysis and critical control points that takes into account the specific features of in situ production, closed habitats, and microbiological monitoring [234]. This represents a shift away from end-product control toward continuous control of the process.

### 11.3. Sensory Acceptability, Menu Fatigue and the Psychological Function of Eating

Space food makes it particularly clear that nutritional value is not enough if the product is not consumed. The review by Taylor et al. notes that astronauts often report altered perceptions of taste and aroma in space, with possible causes including both physiological changes and characteristics of the spacecraft environment: background odours, air and water quality, elevated CO_2_ levels, noise, stress, fatigue, and changes in the context of eating [232]. During a long mission, the issue is not simply whether a product is liked in a sensory panel on Earth, but whether it remains acceptable in a repetitive, confined, and psychologically demanding environment [232,233].

A recent study by Douglas et al. involving ISS astronauts showed that food acceptance and choice have both individual and system-level dimensions. Astronauts evaluated foods during missions lasting approximately six months to one year; the results suggested that acceptance ratings did not decline consistently over time, but crew members tended from the outset to select preferred foods, while many products were rated by only one individual. In a closed system, this can lead to uneven consumption of available items, reduced variety for other crew members, and difficulties in stock planning [233].

Under conditions of restricted choice, sensory acceptability becomes part of nutritional effectiveness: a product with an appropriate composition does not fulfill its intended role if it is not selected and consumed regularly [232,233]. Long-duration systems should assess not only stated product acceptance, but also actual selection patterns and menu diversity.

### 11.4. Resource Constraints and the Transition from Stored Food to Bioregenerative Systems

The current space food system remains largely dependent on Earth: food is designed, produced, controlled, and packaged before a mission and then transported to its destination. This model becomes increasingly demanding for long-duration lunar or Martian missions and future extraterrestrial habitats, where mass, volume, water, energy, storage space, and crew time are critical resources [228,229].

Bioregenerative systems and in situ food production are receiving increasing attention. Johnson et al. described the development of NASA research on plant-based food production as a supplement to packaged food systems and as a step toward bioregenerative life support [239]. Plants can provide fresh food and selected nutrients while potentially contributing to the cycling of oxygen, carbon dioxide, water, and mineral nutrients [229,238,239].

Bioregenerative systems introduce their own resource and safety requirements: they depend on energy, water, cultivation infrastructure, automation, and microbiological control [229,234,239]. In situ production also creates a need to monitor both plant quality and the production environment under conditions where access to a conventional laboratory is limited [234,238,239].

### 11.5. Person-Specific Interpretation of Nutrition in Long-Duration Missions

Long-duration space missions strengthen the need to relate food properties to the response of the individual consumer. Altered physiology, limited dietary variety, potential deficiencies, changes in the microbiota, and the specific conditions of the space environment mean that product composition should not be interpreted independently of the astronaut’s condition [36,235,240]. Research on long-term space nutrition therefore points to the growing importance of a more individualized interpretation of nutritional needs [235,240].

### 11.6. Lessons for Future Food Quality on Earth

Experience from space food systems suggests three principles that can also inform food-quality assessment on Earth: quality should be evaluated over the entire required period of storage and use, not only immediately after production [230,231]; safety should include the resilience and control of the whole process, particularly when food is produced in situ [229,234]; and nutritional value should be interpreted together with actual product acceptability and consumption [232,233]. *Space food* is an extreme but practical model of the *beyond compliance* approach, in which quality means the ability of the food system to retain its function over time and under real conditions of use.

## 12. Personalized Nutrition and Person-Specific Risk Interpretation

Personalization shifts the interpretation of food quality from the product itself to the relationship between the product, the way it is consumed, and the characteristics of the consumer. *Personalized nutrition* is being developed as an approach that uses individual characteristics to formulate dietary recommendations more precisely [241]. Its empirical basis includes both intervention studies and research demonstrating substantial inter-individual variability in responses to food [242,243,244], while the precision nutrition agenda still points to important methodological and implementation gaps [245]. One source of this variability is the gut microbiota, although its potential use in personalization requires cautious interpretation [246,247].

Personalization should not imply a departure from population-based safety standards or the creation of individual legal limits. In this Section, we further develop the concept of *personalized food safety and exposure interpretation* introduced earlier, relating it to consumption patterns, chemical exposure, health status, and biological susceptibility of the consumer [3,29,108].

### 12.1. Inter-Individual Variability in Dietary Responses

One of the best documented examples of inter-individual variability is the difference in metabolic responses to identical or comparable meals. Studies of personalized glycemic responses have shown that models incorporating blood parameters, anthropometric characteristics, physical activity, dietary habits, and the microbiota can predict postprandial glycemic responses more accurately than simply classifying foods according to their average effects [30]. Likewise, findings from the PREDICT 1 study showed marked variability in postprandial glycemic and lipemic responses and only weak correlations between these responses within the same individuals, challenging the assumption that a single metabolic measure is sufficient to describe the body’s response to a meal [243].

This variability reflects overlapping genetic, metabolic, and behavioral factors, including metabolic status, microbiota, sleep, physical activity, and previous dietary patterns [241,243,245]. Personalization should not rely on a single biomarker or the result of one test, but on the integration of multiple characteristics of the individual and the dietary context [241,243,245].

### 12.2. Personalized Nutrition: Evidence, Promise and Limits

The range of data used may include clinical and lifestyle information, genotype, microbiota, and data obtained from digital tools [106,245]. Personalization encompasses a family of approaches that differ in complexity and in the strength of their supporting evidence.

Studies conducted to date suggest that personalization can improve dietary behavior and some health-related outcomes, although the effects are not consistent across all endpoints. In the European Food4Me study, personalized nutritional information was more effective than non-personalized advice in changing selected health-related behaviors [242]. A more recent randomized study by Bermingham et al. showed that a personalized program based on food characteristics, postprandial responses, microbiome data, and health history improved some cardiometabolic outcomes compared with standard dietary advice, although not all parameters differed significantly between groups [244].

The available evidence does not, however, justify presenting precision nutrition as a ready-made solution to all diet-related problems. Bailey and Stover noted that enthusiasm surrounding personalization often runs ahead of the evidentiary standards needed for public-health recommendations [105], while the NIH report continues to identify major needs concerning mechanisms, data quality, research methods, interventions, and implementation of precision nutrition [245].

Most existing precision and personalized nutrition frameworks focus on explaining interindividual differences in dietary response and tailoring dietary advice using phenotypic, clinical, behavioral, genetic, microbiome, and environmental information [105,106,241,245]. The framework proposed here does not replace these approaches or offer an alternative model of personalized nutrition. Its contribution is to extend personalization to the interpretation of food safety and chemical exposure. This requires considering product composition and matrix together with actual consumption, chemical co-exposure, exposure biomarkers, and biological susceptibility [26,29,108,241,245]. The aim is not to construct increasingly detailed individual diets, but to identify situations in which the characteristics of a consumer or consumer group materially change the interpretation of exposure or of an analytical result. This approach therefore complements population-based safety standards and public-health recommendations rather than replacing them [105,245].

### 12.3. Artificial Intelligence and Digital Tools in Personalized Nutrition

Digital tools and AI can support personalization by integrating data on food intake, biomarkers, metabolic responses, and other user characteristics, and by generating individualized recommendations [31,243,245]. The quality of the input data remains critical: image-based and mobile methods, for example, may improve dietary recording, but they are still affected by measurement and interpretation errors [109].

Within the proposed concept of *personalized food safety*, interpretation could also incorporate selected chemical exposures, food–drug interactions, and clinical restrictions [3,110].

### 12.4. Person-Specific Risk: Comorbidities, Drugs and Vulnerable Consumers

Person-specific risk interpretation assumes that the same product composition or level of exposure may have different implications depending on health status, life stage, medication use, and other biological characteristics of the consumer [3,107,241,245]. This becomes particularly evident in chronic disease and polypharmacy, where dietary recommendations need to be interpreted alongside treatment and clinical status. Chronic kidney disease provides a clear example. Recommendations concerning, among others, protein, potassium, phosphorus, sodium, and fluid intake depend on the stage of disease and therapy, while foods and medicines may influence one another in terms of bioavailability, treatment effectiveness, and safety [110]. This shows why product composition cannot always be interpreted independently of the consumer’s clinical context.

### 12.5. Personalized Food Safety and Exposure Interpretation

The concept of *personalized food safety and exposure interpretation* proposed in this manuscript extends classical personalized nutrition to the interpretation of product safety in the context of individual consumption patterns, chemical exposure, and biological susceptibility. The approach draws on the logic of the dietary exposome and on the possibility of relating estimated exposure to biomarkers of exposure [26,29,108]. 

Interpretation may combine four groups of information: data on product composition and matrix; data on the amount and frequency of consumption; consumer characteristics, including health status, microbiota, medications, and biomarkers; and data on exposure and biological response [26,29,108,241]. Integrating these layers does not create individual legal standards, but may help identify when the same product or exposure level should be interpreted differently across consumer groups.

### 12.6. Ethical, Regulatory and Social Challenges

Personalization of nutrition and risk interpretation raises questions about privacy, consent, test quality, responsibility for interpretation, and the transparency of predictive models, because it may involve clinical, genetic, microbiome, and behavioral data [104,106]. A particular concern is the provision of highly precise recommendations based on incomplete data or insufficiently validated models [104,105].

A second issue is equity of access. If advanced personalization is available mainly to selected social groups, it may widen inequalities rather than reduce them [104,105,106,245]. *Personalized nutrition* and *personalized food safety* should complement, rather than replace, population-level safety standards and public-health recommendations [105,245].

### 12.7. Person-Specific Interpretation Within the New Food Quality Compass

Person-specific interpretation provides an operational extension of the personalization dimension within the *New Food Quality Compass*. It does not mean creating separate standards for every consumer, but rather placing compositional and exposure data in the appropriate context of consumption and biological susceptibility. Quality interpretation should specify for which consumer group, at what level of intake, within what dietary context, and with what degree of uncertainty a given product has a particular quality or health significance.

## 13. Public Awareness, Education and Training of Future Food Analysts

The person-specific and uncertainty-aware interpretation developed in the preceding sections depends not only on reliable data, but also on the ability to interpret those data appropriately and use them in practice. On the consumer side, this includes *food literacy* and the ability to use information provided on food labels [248,249,250,251], while communication requires a proper understanding of risk, uncertainty, and numerical information [252,253]. At the professional level, food safety culture and the effective translation of training into practice are equally important [254,255]. This Section focuses on two related levels: consumer education and the competencies of food-quality and food-safety professionals.

### 13.1. Food Literacy, Labels and Informed Consumer Choice

Consumer education should help people interpret product information in the context of portion size, frequency of consumption, and the overall diet rather than frame food choices as a simple distinction between “good” and “bad” foods [248,249,250,251]. *Food literacy* includes both nutritional knowledge and practical skills related to food selection, preparation, and use [248,249]. Labels remain an important source of information, but their value depends on consumers’ ability to interpret them and they should not be treated as a complete description of product quality [250,251].

### 13.2. Risk Communication, Uncertainty and Trust

Within a *food quality under uncertainty* framework, communicating a result is not enough; the source and meaning of uncertainty also need to be explained. EFSA emphasizes the importance of distinguishing missing data, biological variability, model limitations, and limitations in the available evidence [252]. How numerical information is presented also affects understanding; absolute values and natural frequencies may be more useful for communicating risk than relative risk alone [253]. In a food context, risk information should therefore be linked to dose, frequency of consumption, the reference population, and the level of uncertainty [3,75,252,253].

### 13.3. Training Future Food Analysts: From Measurement to Interpretation

A sound analytical foundation remains essential for reliable interpretation. Future analysts need to understand sample representativeness, sample preparation, validation, matrix effects, and measurement uncertainty in accordance with the *fit-for-purpose* approach discussed in Section 9 [169,179,180,182]. Education, however, should not end with the ability to perform an analytical determination correctly.

A laboratory result should be interpreted in the context of method scope, identification confidence, actual consumption, potential exposure, and regulatory relevance [3,56,57,252]. Training should move beyond producing instrument operators toward educating specialists who can judge what a result actually supports—and what it does not.

### 13.4. Interdisciplinary Competence, Data Literacy and Problem-Based Education

Research on food-science education indicates that professional competence extends beyond strictly technical knowledge and includes communication skills, professionalism, decision-making, work in interdisciplinary settings, sustainability, and digital competence [256,257,258,259,260]. Mapping curricula against competency frameworks can help identify gaps between educational content and the expectations of professional practice.

From a *beyond compliance* perspective, this supports wider use of problem-based learning. Such teaching should integrate chromatography, toxicology, food law, and data analysis within tasks that cover sampling, measurement, evaluation of result reliability, exposure interpretation, and regulatory context [256,257,259,260]. Because the analytical applications of chemometrics and artificial intelligence are already discussed in Section 8, the emphasis here is on data and model literacy. Future analysts should understand the difference between exploration, classification, and prediction; recognize overfitting and bias; and be able to judge whether a model has been properly validated and whether it actually addresses the analytical question for which it was developed [165,166,167,259].

### 13.5. Green Analytical Chemistry and Responsible Laboratory Practice

Training in food analysis should also address the environmental and resource implications of laboratory work. Method selection should balance data quality against solvent and energy use, material consumption, time, and cost, with a *fit-for-purpose* approach taking precedence over the assumption that the most complex or sensitive procedure is necessarily the best one [169,195,196,197,198,204]. 

### 13.6. Food Quality and Safety Culture and Lifelong Learning

Technical competence alone is not enough without an organizational culture that supports both safety and quality. Food safety culture involves leadership, organizational responsibility, communication, and the routine application of safety principles in practice [254]. Quality culture extends this perspective to continuous improvement, process stability and reliability, and the use of deviations as a basis for corrective action and further improvement [261]. From a beyond-compliance perspective, the two are closely connected. An effective organizational culture should not only prevent hazards, but also encourage critical interpretation of results, timely responses to emerging signals, and continuous improvement of the quality system. Training is useful only when the knowledge gained can be adapted and applied in professional practice [255]. These competencies therefore need to evolve as hazards, analytical methods, and regulatory requirements change. The resulting competence profile, which combines laboratory skills with data interpretation, exposure assessment, risk communication, and an understanding of the food system, is summarized in Table 6.

## 14. Food Policy and Regulatory Futures

The analytical tools and interpretive competencies discussed in Section 8, Section 9, Section 10, Section 11, Section 12 and Section 13 can influence practice only if they are connected with appropriate regulatory frameworks and food policy. Compliance control should remain the basis of consumer protection, but future food policy will also need broader use of risk-based assessment and risk–benefit analysis [262,263]. From a *beyond compliance* perspective, regulation is not intended to replace existing limits, but to complement them with tools that allow earlier recognition of changes in risk profiles and more adaptive responses to new evidence.

### 14.1. From Limit-Based Control to Risk-Based and Risk-Benefit Governance

Risk-based monitoring should remain the basis for prioritizing official controls. Under EU law, the frequency of official controls is already linked, among other factors, to identified risks, the nature of the activity, and previous compliance history [263]. A further step would be to integrate these data more closely with signals from warning systems and horizon scanning, so that monitoring can also respond to changing patterns of hazards [265,266].

Regulatory monitoring should also make use of results that remain within compliance limits to assess trends, co-occurrence of substances, and the emergence of new analytical signals. The value of monitoring lies not only in identifying exceedances, but also in detecting changes over time in the chemical profile of a product or food category [23,56,170].

A second direction is broader use of *risk–benefit assessment*, particularly where food is simultaneously a source of important nutrients and exposure to contaminants [262]. Fish provide a classic example, as benefits related, among other things, to long-chain n-3 fatty acids must be considered together with exposure to methylmercury, dioxins, PCBs, or PFAS [262]. Where justified, evidence-based regulation should therefore consider both risks and benefits rather than interpret a single parameter in isolation.

### 14.2. Regulatory Implications of Chemical Mixtures and Sub-Threshold Burden

A regulatory consequence of the mixture problem discussed in Section 6 is the need to extend cumulative assessment where there is a sufficient toxicological basis for grouping substances according to a common effect or target organ [3,4,118,119,120]. The concept of *sub-threshold multi-contaminant burden* should not be used to infer harm automatically, but rather to identify products, food categories, and consumption patterns that warrant more detailed monitoring or modeling.

For monitoring, this means reporting not only the proportion of exceedances but also substance co-occurrence, the analytical scope of the method, limits of quantification, and temporal trends [23,56,169,170]. Such data may help prioritize future mixture assessments without replacing toxicological risk assessment with a simple “burden score”.

### 14.3. Early Warning, Foresight and Regulatory Data Infrastructures

Systems such as RASFF and the broader Alert and Cooperation Network enable rapid exchange of information on hazards and non-compliance [265]. The next step, however, is to link reactive *early warning* with horizon scanning and foresight, which are intended to identify weak signals and developments that may affect food safety in the future [37,266,271].

Such a policy requires data infrastructures that allow monitoring, toxicological, and exposure information to be reused and combined. OpenFoodTox is an example of an open, structured toxicological data infrastructure supporting risk assessment [267]. For regulatory data to be useful, however, metadata describing the method, analytical scope, data quality, and uncertainty are equally important; without them, large datasets may remain poorly comparable [56,169].

### 14.4. Harmonization of Methods and Regulatory-Grade Analytical Evidence

As shown in Section 8, modern analytical workflows generate information at different levels of confidence, ranging from a screening signal to a confirmed quantitative result. From a regulatory perspective, it is essential to distinguish clearly between detection, tentative identification, and a result confirmed by a method appropriate to the decision being made [23,56,57].

Data used directly for administrative decisions should come from methods that are appropriately validated and documented for their intended purpose [169]. Harmonization of new tools should not inhibit innovation, but should establish minimum requirements for data quality, identification confidence, and reporting that allow screening results to progress toward evidence of regulatory relevance [23,56,57,169].

### 14.5. Circular Packaging and Chemical Safety

Food-contact materials are an area in which chemical-safety objectives must be considered together with the requirements of a circular economy. The FCM framework requires protection of consumers from substance migration [152], while more recent rules on BPA and other bisphenols strengthen restrictions on selected substances in food-contact materials [268]. Regulation (EU) 2025/40 also reinforces requirements concerning substances of concern, recycling, and reuse of packaging [272]. 

From a regulatory standpoint, recycling and reuse targets should not be pursued independently of migration assessment and realistic use scenarios. NIAS, degradation products, and particle release under actual conditions of material use require particular attention [9,10,143,150]. Environmental objectives in this area need to be aligned with the requirement to maintain the chemical safety of a material throughout its intended life cycle.

### 14.6. Adaptive Regulation of Novel Foods

The regulatory challenge posed by novel foods lies not simply in the emergence of new product categories, but in adapting evidence requirements to the technology, production process, and expected pattern of consumption. Current EFSA guidance for novel food applications includes requirements concerning product characterization, the production process, composition, proposed uses, expected intake, and information needed for safety assessment [153].

Technologies such as cell-based production and precision fermentation raise additional questions concerning biological material, processing, residues, and unexpected products, and EFSA is examining their implications for risk-assessment methodology [270]. Regulation should remain adaptive: it should preserve the requirement for pre-market assessment while allowing evidence requirements to evolve and, where justified, incorporate new data generated after market authorization [34,37,153,270].

### 14.7. Policy Coherence and Transparent Communication Under Uncertainty

A *beyond compliance* approach calls for greater coherence among food safety, public health, and environmental objectives [18,19,264], but this should not mean replacing explicit safety criteria with a single indicator of a “good product.” Safety, nutritional value, and environmental impact should remain distinct and interpretable elements of assessment. Equally important is transparent communication of the basis for regulatory decisions, with a clear distinction between established evidence, signals that warrant further assessment, and situations in which the available data remain insufficient [75,252]. This is especially important in rapidly developing areas such as emerging contaminants and novel foods, where conclusions may change as new evidence becomes available [37,252,266]. Future food governance should therefore combine a strong compliance-control system with risk-based prioritization, foresight, interoperable data, and adaptive evidence requirements.

## 15. Future Research Agenda: What Should Be Measured Now?

Section 15 translates the conceptual arguments developed in the preceding sections into an operational research agenda. The priority is not to identify further categories of uncertainty, but to determine which data should already be collected, modeled, and reported so that future mixture assessment, exposure modeling, analytical screening, and adaptive food-quality management can rely on comparable and reusable information. The priorities below focus on measurable outputs rather than restating problems discussed earlier.

### 15.1. Develop and Validate Multi-Contaminant Burden Metrics

The first priority is to operationalize the concept of *sub-threshold multi-contaminant burden* through the development and validation of screening metrics rather than a single universal risk index. Different ways of describing a multi-contaminant profile should be compared—including the number and classes of detected substances, frequency of co-occurrence, contribution to relevant reference values, and membership in common assessment groups—with existing approaches to cumulative and combined risk assessment [3,4,124,127,128]. 

Any proposed metric should be evaluated for its sensitivity to analytical scope, limit of quantification (LOQ), the weighting of individual components, and assumptions concerning exposure. Results should be reported together with the underlying component profile and uncertainty analysis so that a multidimensional chemical burden is not reduced to an apparently precise single ranking [4,124]. Current studies can already provide the necessary data by reporting the number and classes of detected substances, their concentrations, co-occurrence patterns, and the analytical scope of the method used [124,170].

### 15.2. Link Analytical Data with Real Consumption Patterns

The second priority is the systematic linkage of analytical data with actual consumption. The concentration of a substance in a product should be interpreted together with portion size, frequency of consumption, and the structure of the overall diet. Total Diet Studies provide an important model for generating exposure data from foods analyzed in a form close to that actually consumed [26,273].

Exposure models should report not only mean values but also exposure distributions and high-consumption scenarios. Analytical data need to be stored in a structure that permits linkage with food codes, population groups, consumption scenarios, and the method used to calculate exposure [273,274]. Interoperability and reuse of such datasets should be supported by the Findable, Accessible, Interoperable, and Reusable (FAIR) principles [275].

### 15.3. Pilot Structured Suspect and Non-Target Screening in Routine Monitoring

The third priority is the pilot implementation of suspect and non-target screening within structured monitoring as a layer complementing targeted analysis rather than automatically replacing it. Implementation studies should define priority lists, quality assurance/quality control (QA/QC) procedures, criteria for signal prioritization, and conditions that trigger confirmatory analysis [134,174,175].

Equally important is the archiving of HRMS data, analytical feature lists, metadata, and identification confidence levels so that samples can be analyzed retrospectively when new libraries or toxicological signals become available [56,174,175]. Future studies should focus particularly on reproducibility, transferability between laboratories, the proportion of signals requiring confirmation, and the value of retrospective data re-interrogation [56,174,175].

### 15.4. Design Realistic Low-Dose Mixture Experiments from Occurrence Data

The fourth priority is to design mixture experiments using actual co-occurrence and exposure data. At least two complementary approaches are useful: mixtures constructed around a common mechanism or target organ, and mixtures reconstructed from real co-occurrence profiles found in foods and diets [3,124,127,128].

Component ratios, dose ranges, and exposure duration should reflect monitoring data and realistic consumption scenarios. Reporting should include assumptions about additivity, possible deviations from it, the biological model used, uncertainty in extrapolation, and sensitivity analysis [6,7,124]. Early mechanistic data may help prioritize mixtures for further study, but they should not automatically be interpreted as evidence of human risk.

### 15.5. Measure Migration and Transformation from Food-Contact Materials Under Real-Use Conditions

The fifth priority is to investigate migration and substance release under conditions that reflect actual use of food-contact materials. Study designs should account for factors that may alter migration, such as heating, prolonged contact, repeated use, mechanical wear, and material aging, and should combine targeted analysis with investigation of NIAS and, where relevant, particle release [102,103,143,150].

For reusable and recycled materials, reporting the history of the material and test conditions is particularly important. Unknown signals should be prioritized according to reproducibility, migration level, frequency of occurrence, and available toxicological information [9,26,143,151]. Where possible, migration results should be related to actual human exposure rather than interpreted solely on the basis of the presence of a substance in the material [9,143,151].

### 15.6. Define Pre-Scale-Up Evidence Packages for Future Foods

The sixth priority is to define technology-specific evidence packages required before the broad scale-up of future foods. A core dataset should include characterization of composition and nutritional value, allergenicity, microbiological and storage stability, process residues or metabolites, and the expected pattern and level of consumption [34,153,270].

For cell-based production and precision fermentation, additional characterization is needed for the biological material, critical process elements, purification, batch stability, and the potential formation of unexpected products [270]. The research agenda should then link pre-scale-up data with predefined post-market monitoring points where significant uncertainties remain [34,37].

### 15.7. Quantify When Person-Specific Information Changes Exposure Interpretation

The seventh priority is to determine quantitatively when person-specific information genuinely changes exposure interpretation or dietary decisions compared with a population-based model. Studies should define relevant population strata in advance, assess the added value of clinical information, microbiota, or biomarkers, and report whether their inclusion leads to a materially different classification of exposure or recommendation [241,245,246]. Within personalized food safety, separate attention should also be given to scenarios involving supplements and functional foods, where dose, food–drug interactions, and the safety of ingredients without a long history of consumption may be important [276]. The aim is not to create individual legal limits, but to identify situations in which personalization provides measurable value beyond standard population-level assessment.

### 15.8. Define a Minimum Reporting Package for Future Food Quality Studies

The eighth priority is to develop a modular *minimum reporting package* for studies of food quality under uncertainty. The core module should include product and matrix identification, origin and sampling time, sample preparation, analytical method, validation scope, QA/QC, limit of detection (LOD)/LOQ, uncertainty, and the range of analytes covered by the method [169,274].

Study-specific information should be added to this core module. In suspect/NTS studies, this should include feature-processing procedures, identification confidence level, libraries used, blank correction, and confirmation status [56,175]; in exposure studies, it should include the food-classification system, assumptions concerning consumption, population exposure percentiles, and the method used to calculate exposure [273,274].

Data and metadata should be prepared in accordance with the FAIR principles so that they can later be found, integrated, and reused [275]. Limitations should be reported with equal transparency, including compound classes not covered by the method, inadequate LOQs, low-confidence identifications, and the absence of consumption data for relevant population groups [56,175,274].

### 15.9. Evaluate Training Outcomes and Foresight Capacity

The competency profile of future food analysts was discussed in detail in Section 13; within the research agenda, the more important question is whether academic curricula and professional training actually reduce the identified competency gaps. Studies may use curriculum mapping, assessment of performance in problem-based tasks, and comparison of the ability to interpret and communicate ambiguous results before and after training [256,257,260]. Foresight can complement such evaluation by examining whether institutions identify emerging issues early enough and adapt monitoring and professional competencies accordingly [266].

Together, these priorities form an operational research agenda centered on data that can be generated now and reused as analytical, toxicological, and regulatory frameworks continue to evolve. The principal elements of measurement, modeling, and reporting are summarized in Table 7.

## 16. Conclusions

This review shows that contemporary food quality cannot be fully described either by a single product parameter or by compliance with legal requirements alone. Microbiological and chemical control, contaminant limits, validated analytical methods, and official control remain essential foundations of consumer protection. The *beyond compliance* approach is not intended to replace them, but to complement them where quality also depends on the food matrix, processing, co-occurrence of substances, actual consumption patterns, consumer characteristics, conditions of product use, and a changing food system. From this perspective, quality becomes a dynamic profile interpreted in terms of benefits, risks, and uncertainty.

The first of the three conceptual outcomes of this review is *food quality under uncertainty*. This framework draws attention to the fact that the chemical space of food is not fully characterized, the effects of all mixtures cannot be predicted directly, average consumption does not always represent the actual exposure of specific population groups, and some emerging technologies are developing faster than the analytical, toxicological, and regulatory tools used to assess them. The concept of *sub-threshold multi-contaminant burden* extends this issue to recurrent co-exposure to multiple compounds present at low levels. It does not imply that the presence of many substances below their individual limits is itself evidence of harm. Rather, it supports the development and validation of metrics describing co-occurrence, exposure, and uncertainty, and the identification of situations that require more detailed mixture assessment.

The third conceptual outcome is the *New Food Quality Compass*, which organizes food quality across interconnected dimensions of safety, nutritional value, bioactivity, matrix, processing, contaminants, authenticity, sustainability, personalization, and trust. The Compass is neither an algorithm for classifying products as “good” or “bad” nor a single quality index. Its value lies in making visible the trade-offs and interpretive gaps between different dimensions of quality. It allows a product to be assessed without reducing its quality to a single technological, nutritional, or marketing category.

The analytical consequence of this perspective is a shift from asking only “what was detected?” to asking about method scope, identification confidence, sample representativeness, magnitude of exposure, and the relevance of the result to a particular consumer or population. Targeted analyses remain the basis of quantitative compliance control, while *suspect* and *non-target screening*, foodomics, exposomics, and HRMS data archiving can extend the capacity to detect and retrospectively assess unexpected signals. The usefulness of these tools nevertheless depends on reliable sample preparation, appropriate QA/QC, transparent reporting of identification confidence, and linkage of compositional data with actual consumption. Person-specific interpretation should likewise complement, rather than replace, population-level safety standards; its value should be judged by whether the inclusion of clinical, biological, or behavioral information genuinely changes exposure interpretation or recommendations.

Future development of food quality and safety assessment should focus less on increasing the number of measured parameters and more on generating information that can change exposure interpretation or decision-making. Immediate priorities include reporting contaminant co-occurrence profiles together with the analytical scope of the method, linking analytical results with actual consumption data, introducing structured suspect and non-target screening into monitoring programs, investigating realistic mixtures and migration from food-contact materials under real-use conditions, and defining minimum evidence packages for new food sources before large-scale implementation. Over the longer term, multi-contaminant exposure metrics will need further validation, and the added value of person-specific information should be tested against whether it genuinely improves risk interpretation. Transparent reporting of uncertainty, interoperable data, and data reuse remain important across all of these areas. Progress will also depend on specialists who can recognize the limitations of measurements, interpret uncertainty, and translate analytical findings into toxicological, nutritional, technological, and regulatory terms. *Beyond compliance* does not replace compliance control. It identifies where additional information is needed when compliance alone is not sufficient to characterize food quality and safety.

## Figures and Tables

**Table 1:** Search strategy and inclusion criteria.

Component	Proposed Specification
Review type	Narrative and conceptual synthesis supported by structured search
Main databases	Web of Science, Scopus and PubMed/MEDLINE as main databases; ScienceDirect and Google Scholar as supplementary sources
Time frame	Mainly 2000–2026; older works retained if historically or methodologically foundational
Keyword blocks	Food quality/safety; contaminants/exposure; processing categories; analytical tools; future foods/personalization
Inclusion criteria	Peer-reviewed reviews and original papers, methodological papers, EFSA/FAO/WHO documents, high-quality institutional reports
Exclusion criteria	Marketing material, unclear authorship, insufficient methods, weak relevance, untraceable gray literature
Evidence organization	Thematic grouping rather than purely chronological listing

**Table 2:** Comparative quality profiles, trade-offs and monitoring priorities across selected food categories.

Food Category	Potential Quality Strengths	Potential Limitations/Trade-Offs	What Should Be Measured and Reported Now	Refs.
Minimally processed foods	Freshness, preserved matrix, fewer additives	Microbiological vulnerability, short shelf life, variable raw material quality	Cold-chain data, microbial indicators, storage time, toxins, metals, pesticide residues, matrix integrity	[63,77,78,79,80]
Traditional foods	Cultural identity, sensory value, trust, local heritage	Variable processes, inconsistent safety control, possible high salt, alcohol or biogenic amines	Process parameters, fermentation stability, microbial safety, salt/alcohol/amine profile, origin verification	[81,82,83,84,85]
Organic foods	Lower synthetic chemical input, consumer trust, certain environmental benefits	Not automatically safer or more nutritious; yield and context matter	Residue frequency, natural toxins, microbial quality, cadmium/metals, environmental indicators	[86,87,88,89,90,91]
Conventional foods	Productivity, standardization, affordability, stable supply	Intensification costs, residues, soil/water pressure, biodiversity and emission pressures	Residue trends, fertilizer/pesticide use, traceability, soil/water pressure and other environmental indicators	[20,40,41,89,90,91]
Processed foods	Safety, shelf life, convenience, fortification, reduced waste	Matrix transformation, nutrient losses, process contaminants, packaging exposure	Processing parameters, neoformed compounds, nutrient stability, packaging migration, portion context	[12,40,62,92,102,103]
Ultra-processed foods	Convenience, palatability, availability, price stability	High energy density, altered matrix, additive complexity, overconsumption risk	NOVA category, ingredient markers, energy density, additives, texture, eating rate, diet-level exposure	[15,16,17,93,96,97,98,99,100,101]
Functional foods	Targeted bioactivity and health-oriented positioning	Composition does not equal demonstrated health effect	Bioaccessibility, bioavailability, effective dose, clinical evidence level, population specificity	[65,66,67]
Personalized foods/nutrition	Adaptation to individual needs and responses	Evidence gaps, data privacy, algorithmic opacity, inequity	Response biomarkers, microbiome context, comorbidities, medication interactions, data governance	[30,31,104,105,106,107,108,109,110]
Future and space foods	Sustainability, resilience, resource efficiency, novel matrices	Allergenicity, process metabolites, scale-up uncertainties, acceptance, closed-system risks	Nutritional adequacy, novel hazards, process residues, stability, sensory acceptance, foresight signals	[32,33,34,35,36,37,111,112]

**Table 3:** Emerging contaminants and blind spots in food quality control.

Emerging Contaminant/Blind Spot	Reason for Limited Coverage	Priority Matrices/Pathways	Main Challenge	Monitoring Priority	Refs.
PFAS	Persistent and mobile chemicals with dietary and global trade relevance	Fish, seafood, animal products, water, packaging-related pathways	Large compound class, changing limits, mixture exposure, source attribution	Targeted PFAS panels, precursors, origin, co-exposure with diet patterns	[11,113,133,135,137,138]
Microplastics and nanoplastics	Particles are difficult to define, quantify and link to effects	Bottled water, seafood, salt, packaging, food-contact materials	Sampling contamination, size fractions, polymer ID, mass vs particle number	Standardized blanks, polymer type, particle size, shape, migration scenarios	[10,114,139,140,142,150]
Food-contact chemicals and NIAS	Many migrants are not intentionally added or fully characterized	Plastics, paper/board, recycled materials, coatings, adhesives, inks	Unknown compounds, mixtures, degradation products, real-use migration	Targeted + NTS migration tests, exposure scenarios, toxicity prioritization	[8,9,102,103,143,151,152]
Pesticide metabolites/transformation products	Parent compounds may not describe total pesticide-related exposure	Fruits, vegetables, grains, processed products, soil-derived matrices	Reference standards lacking, unknown toxicity, formation during processing/storage	Parent-metabolite panels, transformation products, cumulative groups	[4,149]
Climate-sensitive mycotoxins	Climate change can shift fungal ecology and toxin geography	Cereals, nuts, fruits, spices, animal feed, milk carry-over	Co-occurrence, modified forms, predictive modeling, seasonal variability	Multi-mycotoxin panels, modified forms, climate/geographic metadata	[21,129,130,136]
Process contaminants	Safety can be affected by useful preservation/cooking technologies	Thermally processed, fried, smoked, dried or refined products	Balance between microbiological safety and chemical formation	Acrylamide-like markers, furanic compounds, PAHs, MCPDs	[92,145,146]
Mineral oil hydrocarbons	Complex mixtures from packaging, lubricants and processing environment	Dry foods, cereal products, recycled cardboard, oils/fats	MOSH/MOAH fraction interpretation and toxicological uncertainty	Fraction profiles, packaging source, storage and contact conditions	[147,148]
Novel food ingredients/technologies	Composition and exposure may change with new production processes or matrices	Novel ingredients, precision-fermentation products, cell-based ingredients and technologically designed foods	Composition, process-derived residues, allergenicity, stability and exposure assessment	Identity, composition, process residues, stability, intended use and exposure	[34,37,153]

**Table 4:** Analytical tools for future food quality assessment.

Tool/Workflow	Information Generated	Strengths	Limitations	Best Role in Future Quality Assessment	Refs.
Targeted LC-MS/MS/GC-MS/MS	Quantitative data for predefined analytes	High selectivity and quantitative performance	Limited to predefined analytes and available standards	Compliance monitoring and confirmatory analysis	[154,155,157]
LC/GC-HRMS	Accurate mass data for targeted, suspect and non-target workflows	Detects unexpected signals and retrospectively mines data	Identification confidence, libraries, quantification and validation remain challenging	Emerging risk screening and discovery-oriented monitoring	[23,56,134]
ICP-MS	Elemental contaminants and trace elements	Excellent sensitivity for metals and elements	Speciation often requires additional methods	Metals, metalloids and elemental exposure assessment	[176]
NMR	Non-destructive structural and fingerprint information	Highly reproducible and useful for authenticity/metabolomics	Lower sensitivity than MS for trace contaminants	Foodomics, authenticity and matrix-level profiling	[157,159]
FTIR/Raman	Molecular fingerprints and rapid classification	Fast, non-destructive, low sample consumption	Often indirect; requires chemometrics and calibration transfer	Screening, authenticity, process and matrix monitoring	[162,167]
SERS	Enhanced Raman signals for trace targets	High sensitivity and potential portability	Substrate reproducibility and validation issues	Rapid screening of selected hazards or authenticity markers	[163,164]
Hyperspectral imaging	Spatially resolved spectral fingerprints	Non-destructive, useful for heterogeneity and defects	High data volume, model transfer and calibration challenges	Screening of quality, contamination, sorting and defects	[161,162]
Chemometrics and AI	Pattern recognition, classification, prediction, anomaly detection	Integrates large multidimensional datasets	Black-box risk, bias, need for validation and explainability	Decision support, prioritization and trend detection	[158,165,166,167,177]

Abbreviations: LC-MS/MS, liquid chromatography-tandem mass spectrometry; GC-MS/MS, gas chromatography-tandem mass spectrometry.

**Table 5:** Sample preparation bottlenecks and future directions.

Stage/Bottleneck	Main Problem	Future Direction	Minimum Reporting Requirement	Refs.
Sampling and representativeness	Food is heterogeneous across units, batches and supply chains	Risk-based sampling plans and metadata-rich sampling protocols	Sampling frame, number of units, mass, location, batch, date	[179,180]
Homogenization	Incomplete size reduction or phase separation	Matrix-specific homogenization and cryogenic/controlled protocols	Equipment, time, temperature, particle size where possible	[180,198]
Stabilization and storage	Analytes can degrade, transform or migrate during storage	Validated holding times and stabilization conditions	Storage time, temperature, container, freeze–thaw history	[180,198]
Extraction	Different analyte classes require different polarity and conditions	Multi-class or modular extraction workflows	Solvent, pH, time, temperature, ratio, extraction energy	[184,185,186,187,194]
Clean-up	Matrix removal can also remove relevant analytes	Balanced cleanup and matrix-matched validation	Sorbents, cleanup steps, recovery for representative analytes	[53,181,193,194,198]
Matrix effects	Co-extractives suppress or enhance signals	Internal standards, matrix matching, standard addition or dilution strategies	Matrix-effect evaluation and correction approach	[181,182,183]
QuEChERS/micro-QuEChERS	Flexible but not universal for all matrices and analytes	Fit-for-purpose optimization and miniaturization	Scope, recovery range, validation level, excluded analyte classes	[53,54,55,193,195]
Microextraction and sorbent approaches	High selectivity can be both strength and bias	Tailored sorbents and transparent selectivity reporting	Sorbent type, capacity, reuse, selectivity, breakthrough if relevant	[188,198,199,200,201]
Green solvents and energy-assisted extraction	Reduced environmental burden may be accompanied by analytical trade-offs	Green metrics combined with validation and uncertainty	Solvent toxicity, volume, energy, recovery, precision	[186,187,195,197,204]
Automation and high throughput	Automation can reproduce systematic bias across large sample series	Robotic workflows with QC checkpoints and blanks	QC sample frequency, blanks, carryover, batch correction	[205]
Fit-for-purpose decision	One method cannot serve all analytical questions	Define whether method is screening, confirmatory, exploratory or regulatory	Intended use, validation depth, uncertainty, limitations	[169,198,204]

**Table 6:** Future competencies for food analysts and food safety professionals.

Competence Domain	Why It Matters	Suggested Learning Outcomes	Refs.
Classical food analysis	Still provides the foundation of compliance and quality control	Select validated methods, understand accuracy, precision, uncertainty and fit-for-purpose criteria	[169,179,180]
Sample preparation	Determines representativeness, recovery and matrix effects	Design sampling, homogenization, extraction and cleanup for complex matrices	[53,54,55,179,180,198]
Mixture toxicology	Future risk assessment increasingly concerns co-exposure	Explain additivity, synergy, antagonism and cumulative assessment groups	[3,4,5,6,7]
Exposure assessment	Product composition must be linked to real diets	Use consumption data, vulnerable groups and probabilistic thinking	[3,26,115,116]
Foodomics and NTS	Expands beyond known analytes and single markers	Interpret HRMS levels of identification, fingerprints and omics outputs	[24,25,56,57]
Data literacy and model validation	Multidimensional analytical and surveillance data require critical model assessment	Recognize overfitting and bias; validate, interpret and critically assess models	[165,166,167,259]
Food law, policy and regulatory evidence	Analytical data acquire meaning in regulatory context	Interpret official controls, risk-benefit guidance, regulatory databases, food-contact rules and novel-food assessment frameworks	[262,263,264,265,266,267,268,269,270]
Green analytical chemistry	Laboratories must reduce solvent, energy and waste burden	Apply miniaturization, safer solvents and sustainability metrics	[195,196,197,203,204]
Risk communication	Trust depends on transparent uncertainty communication	Explain knowns, unknowns, uncertainty and practical implications without alarmism	[75,252,253]
Food safety foresight	Emerging risks require anticipatory thinking	Identify signals from climate, trade, technologies and diets	[34,37,266]

**Table 7:** Operational research priorities and recommended measurements, modeling, and reporting.

Research Priority	What Should Be Measured Now	What Should Be Modeled/Reported	Why It Matters	Refs.
Multi-contaminant burden metrics	Co-occurring contaminant number, classes, concentrations and product/diet profiles	Candidate metrics, co-occurrence matrices, uncertainty and sensitivity analyses	Moves beyond single-limit compliance without claiming automatic harm	[3,4,124,127,128]
Real consumption and exposure	Food composition; portion size; consumption frequency; preparation context; population-specific intake	Dietary exposure distributions and high-consumer scenarios	Connects analytical data with real-life risk interpretation	[26,273,274]
Structured suspect/NTS monitoring	HRMS features; suspect matches; identification confidence; archived analytical data	Prioritization lists, confirmation status and trend signals	Supports detection of unexpected chemical signals before routine targets exist	[56,134,174,175]
Low-dose mixture experiments	Mixture composition; component ratios; diet-relevant dose levels; exposure duration	Dose–response patterns; additivity and deviations from additivity; shared targets or pathways; mechanistic interaction evidence; sensitivity analyses	Tests the adequacy of additivity-based assumptions for diet-relevant mixtures	[3,6,7,124]
Food-contact migration	Targeted migrants; NIAS; degradation products; particle release under real-use conditions	Migration scenarios, repeated-use, heating, wear and aging effects	Captures hidden exposure from packaging, recycled and reusable materials	[9,102,103,143,150]
Future foods before scale-up	Nutritional composition; allergens; residues; process metabolites; stability; intended use and exposure	Pre-market evidence packages and predefined post-market monitoring triggers	Supports evidence generation before broad scale-up and targeted follow-up where uncertainty remains	[34,153,270]
Person-specific exposure interpretation	Population strata; clinical variables; microbiome characteristics; exposure and response biomarkers	Incremental value over population-level exposure models	Identifies when personalization materially changes exposure or dietary interpretation	[241,245,246,276]
Minimum reporting package and FAIR data	Analytical metadata, QA/QC, LOD/LOQ, uncertainty, method scope	Reusable datasets linked to toxicology and consumption data	Turns isolated measurements into reusable and integrable evidence	[267,274,275]
Training outcomes and foresight capacity	Curriculum coverage; competency gaps; integrative-task performance; foresight indicators	Pre/post-training outcomes, curriculum mapping and institutional response to emerging signals	Tests whether training improves professional competence and institutional foresight in practice	[256,257,260,266]

## Data Availability

No new data were created or analyzed in this study. Data sharing is not applicable to this article.

## Abbreviations

AI, artificial intelligence; EFSA, European Food Safety Authority; FAIR, Findable, Accessible, Interoperable, and Reusable; FAO, Food and Agriculture Organization of the United Nations; GC-HRMS, gas chromatography-high-resolution mass spectrometry; HRMS, high-resolution mass spectrometry; ISS, International Space Station; LC-HRMS, liquid chromatography-high-resolution mass spectrometry; LC-MS/MS, liquid chromatography-tandem mass spectrometry; LOQ, limit of quantification; MCRA, Monte Carlo Risk Assessment; MOAH, mineral oil aromatic hydrocarbons; MOSH, mineral oil saturated hydrocarbons; MRL, maximum residue limit; NIAS, non-intentionally added substances; NTS, non-target screening; PFAS, per- and polyfluoroalkyl substances; PRISMA, Preferred Reporting Items for Systematic Reviews and Meta-Analyses; QA/QC, quality assurance/quality control; QuEChERS, quick, easy, cheap, effective, rugged, and safe; SERS, surface-enhanced Raman spectroscopy; SPE, solid-phase extraction; WHO, World Health Organization.
